# Coordinate systems for supergenomes

**DOI:** 10.1186/s13015-018-0133-4

**Published:** 2018-09-24

**Authors:** Fabian Gärtner, Christian Höner zu Siederdissen, Lydia Müller, Peter F. Stadler

**Affiliations:** 1 Competence Center for Scalable Data Services and Solutions Dresden/Leipzig, Universität Leipzig, Augustusplatz 12, 04107 Leipzig, Germany; 2 Bioinformatics Group, Department of Computer Science, Universität Leipzig, Härtelstraße 16–18, 04107 Leipzig, Germany; 3 Interdisciplinary Center for Bioinformatics, Universität Leipzig, Härtelstraße 16–18, 04107 Leipzig, Germany; 4 Automatic Language Processing Group, Department of Computer Science, Universität Leipzig, Augustusplatz 12, 04107 Leipzig, Germany; 5 Max Planck Institute for Mathematics in the Sciences, Inselstraße 22, 04103 Leipzig, Germany; 6 Department of Theoretical Chemistry, University of Vienna, Währinger Straße 17, 1090 Vienna, Austria; 7 Center for non-coding RNA in Technology and Health, Grønegårdsvej 3, 1870 Frederiksberg C, Denmark; 8 Santa Fe Institute, 1399 Hyde Park Rd., Santa Fe, NM 87501 USA

**Keywords:** Comparative genomics, Comparative transcriptomics, Big data, Graph theory, Betweenness ordering, Colored multigraph, Combinatorial optimization

## Abstract

**Background:**

Genome sequences and genome annotation data have become available at ever increasing rates in response to the rapid progress in sequencing technologies. As a consequence the demand for methods supporting comparative, evolutionary analysis is also growing. In particular, efficient tools to visualize-omics data simultaneously for multiple species are sorely lacking. A first and crucial step in this direction is the construction of a common coordinate system. Since genomes not only differ by rearrangements but also by large insertions, deletions, and duplications, the use of a single reference genome is insufficient, in particular when the number of species becomes large.

**Results:**

The computational problem then becomes to determine an order and orientations of optimal local alignments that are as co-linear as possible with all the genome sequences. We first review the most prominent approaches to model the problem formally and then proceed to showing that it can be phrased as a particular variant of the Betweenness Problem. It is NP hard in general. As exact solutions are beyond reach for the problem sizes of practical interest, we introduce a collection of heuristic simplifiers to resolve ordering conflicts.

**Conclusion:**

Benchmarks on real-life data ranging from bacterial to fly genomes demonstrate the feasibility of computing good common coordinate systems.

**Electronic supplementary material:**

The online version of this article (10.1186/s13015-018-0133-4) contains supplementary material, which is available to authorized users.

## Background

The past decade has seen rapid progress of sequencing technologies [1]. The dramatic decrease of sequencing costs has enabled an ever-accelerating flood of genomic and transcriptomic data [2] that in turn have lead to the development of a wide array of methods for data analysis. Despite recent efforts to study transcriptome evolution at large scales [3–7] the capability to analyze and integrate -omics data in large-scale phylogenetic comparisons lags far behind data generation. One key aspect of this shortcoming is the current lack of powerful tools for visualizing comparative -omics data. Available tools such as [8, 9] have been designed with closely related species or strains in mind. The visualizations become difficult to read for multiple species and larger evolutionary distances, where homologous genomic regions may differ substantially in their lengths, an issue that becomes more pressing the larger regions of interest become. A common coordinate system for multiple genomes is not only a convenience for graphical representations of -omics data, however. It would also greatly facilitate the systematic analysis of all those genomic features that are not sufficiently local to be completely contained within individual blocks of a genome-wide multiple sequence alignment (gMSA).

Still, gMSAs are the natural starting point. Several pipelines to construct such alignments have been deployed over the past two decades, most prominently the tba/multiz pipeline [10, 11] employed by the UCSC genome browser and the Enredo/Pecan/Ortheus (EPO) pipeline [12] featured in the ensembl system. For the ENCODE project data, in addition alignments generated with MAVID [13] and M-LAGAN [13] have become available, see [14] for a comparative assessment. A common feature of gMSAs is that they are composed of a large number of alignment blocks. At least in the case of MSAs of higher animals and plants the individual blocks are typically (much) smaller than individual genes. As a consequence, they are not ready-to-use for detailed comparative studies e.g. of transcriptome or epigenome [15] structure. In the gMSA-based splice site maps of [16], for example, it is easy to follow the evolution of individual splice junctions as they are localized within a block. At the same time it is difficult to collate the global differences of extended transcripts, which may span hundreds of blocks and to relate changes in transcript structure with genomic rearrangements, insertions of repetitive elements or deletion of chunks of sequence.

To a certain extent this problem is alleviated by considering the blocks arranged w.r.t. a reference genome. For many applications, however, this does not appear to be sufficient. For sufficiently similar genomes with only few rearrangements gMSA blocks are large or can at least be arranged so that large syntenic regions can be represented as a single aligned block. Any ordering of these large syntenic blocks, termed a supergenome in [17], then yields an informative common coordinate system. So far, this approach has been applied only to closely related procaryotic genomes. Prime examples are a detailed comparative analysis of the transcriptome of multiple isolates of *Campylobacter jejuni* [18] or the reconstruction of the phylogeny of mosses from the “nucleotide pangenome” of mitogenomic sequences [19]. We remark that some approaches to “pangenomes” are concerned with gMSAs of (usually large numbers of) closely related isolates; most of this literature, however, treats pangenomes as sets of orthologous genes [20].

Here we are concerned with the coordinatization of supergenomes, i.e., the question how gMSA blocks can be ordered in a way that facilitates comparative studies of genome annotation data. In contrast to previous work on supergenomes we are in particular interested in large animal and plant genomes and in large phylogenetic ranges. We therefore assume that we have short alignment blocks and abundant genome rearrangement, leaving only short sequences of alignment blocks that are perfectly syntenic between all genomes involved. The problem of optimally sorting the MSA blocks can, as we shall see, be regarded as a quite particular variant of a vertex ordering problem, a class of combinatorial problems that recently has received increasing attention in computer science [21–24]. In the computational biology literature, furthermore, several graph-based methods have been proposed to solve the problem of sorting sequence blocks for supergenomes, see e.g. [12, 25–29].

This contribution is organized as follows: In the following section we first analyze the concept of the supergenome and its relationship to gMSAs in detail. We then review combinatorial optimization problems that are closely related to the “supergenome sorting problem”, and argue that the most appropriate modeling leads to a special type of betweenness ordering problem. Next, we introduce a heuristic solution that is geared towards very large input alignments and proceeds by step-wise simplification of the supergenome multigraph. Finally, we outline a few computational results.

## Theory

### Genome-wide multiple sequence alignments

Our starting point is a set of genome assemblies. For our purposes an assembly is simply a set of sequences representing chromosomes, scaffolds, reftigs, contigs, etc. In the following, we will use *contig* to refer to any such sequence. On each of these constituent sequences we assume the usual coordinate system defining sequence positions. Since DNA is double stranded, a piece of genomic sequence is either contained directly ($\sigma = +\;1$) in the assembly or it is represented by its reverse complement ($\sigma = -\;1$). We write $(\mathcal {G},c,i,j,\sigma)$ to identify the *sequence interval* from positions *i* to *j* on contig *c* of genome assembly $\mathcal {G}$ with reading direction $\sigma$. We assume, w.l.o.g., $i\le j$.

Most comparative methods require multiple sequence alignments (MSAs) as input. An MSA $\mathfrak{A}$ is composed of *alignment blocks*, each of which consists of an alignment of sequence intervals. For the purposes of this paper it its sufficient to characterize an alignment block by the coordinates of its constituent sequence intervals. That is, a block $B\in \mathfrak{A}$ has the form $B=\{ (\mathcal {G}_u,c_u,i_u,j_u,\sigma _u) | u \in \text {rows of }B\}$ where the index *u* runs over the rows of the alignment block. It will be convenient to allow alignment blocks also to consist of a single interval only, thus referring to a piece of sequence that has not been aligned. Note that at this stage we do not assume that an alignment block contains only one interval from each assembly.

The projection $\pi _\mathcal {G}(B)$ extracts from an alignment block the union of its constituent sequence intervals belonging to assembly $\mathcal {G}$. If the assembly $\mathcal {G}$ is not represented in the alignment block *B* we set $\pi _\mathcal {G}(B)=\emptyset$.

The projection operation collapses pairs of overlapping sequence intervals ($i \le i' \le j \le j'$) in to a single interval: $(\mathcal {G},c,i,j,\sigma)\cup (\mathcal {G},c,i',j',\sigma ')= (\mathcal {G},c,i,j',+1)$ without regard for the orientation, which is set to $+1$ and will have *no bearing* on the algorithm we develop further down.

The projection $\pi _\mathcal {G}$ of $\mathfrak{A}$ onto one of its constituent assemblies $\mathcal {G}$ is the union of the sequence intervals from $\mathcal {G}$ that are contained in its alignment blocks, i.e., $\pi _\mathcal {G}(\mathfrak{A})=\bigcup _{B\in \mathfrak{A}} \pi _\mathcal {G}(B)$.

#### **Definition 1**

Let $\mathfrak{A}$ be an MSA.

- $\mathfrak{A}$ is *complete* if $\pi _\mathcal {G}(\mathfrak{A})=\mathcal {G}$, i.e., if each position in each assembly is represented in at least one alignment block.
- $\mathfrak{A}$ is *irredundant* $\pi _\mathcal {G}(B')\cap \pi _\mathcal {G}(B'')=\emptyset$ for any two distinct blocks $B'$ and $B''$, i.e., if every sequence interval from assembly $\mathcal {G}$ is contained in at most one alignment block.
- $\mathfrak{A}$ is *injective* if no alignment block comprises more than one interval from each of its constituent assemblies.

Clearly, every given MSA can easily be completed by simply adding all unaligned sequence intervals as additional blocks.

Just like a contig *c* in a (genome) assembly $\mathcal {G}$, each block $B\in \mathfrak{A}$ has an internal coordinate system defined by its columns. We write (*B*, *k*) for column *k* in block *B*. We write $\ell (B)$ for the number of columns in block *B*. If $\mathfrak{A}$ is irredundant, then there are functions $f_{\mathcal {G},c}$ that map position *i* within $(\mathcal {G},c)$ to a corresponding MSA coordinate (*B*, *k*). If $\mathfrak{A}$ is complete, the individual $f_{\mathcal {G},c}$ can be combined to a single function $f:(\mathcal {G},c,i)\mapsto (B,k)$. Completeness implies that every position $(\mathcal {G},c,i)$ is represented in the MSA, and irredundancy guarantees that the relation between assembly and alignment coordinates is a function by ensuring that $(\mathcal {G},c,i)$ corresponds to at most one alignment column. The following definition is therefore equivalent to the notion of a supergenome introduced in [17].

#### **Definition 2**

An MSA $\mathfrak{A}$ is a *supergenome* if it is complete, irredundant, and injective.

The most commonly used genome-wide MSAs cannot be completed to supergenomes. The MSAs produced by the multiz pipeline are usually not irredundant: different intervals of the “reference sequence” may be aligned to the same interval of another assembly. While multiz [11] alignments are injective this is in general not the case with the EPO [12] alignments. In these, multiple paralogous sequences from the same genome may appear in one alignment block.

Now consider an MSA $\mathfrak{A}$ and an arbitrary order < of the alignment blocks of $\mathfrak{A}$. Then there is a (unique) function $\phi$ that maps the pairs (*B*, *k*) injectively to the interval [1, *n*], where $n=\sum _{B\in \mathfrak{A}} \ell (B)$ is the total number of columns in $\mathfrak{A}$ such that $\phi (B,i)<\phi (B',i')$ whenever $B<B'$ or $B=B'$ and $i<i'$. If $\mathfrak{A}$ is a supergenome, then $\phi (f)$ is clearly an injective function from a genome assembly $\mathcal {G}$ to [1, *n*]. We call $\phi (f(\mathcal {G},c,i))$ the *coordinate* of position *i* of contig *c* of assembly $\mathcal {G}$ in the ordered supergenome $(\mathfrak{A},<)$.

As pointed out in [17], the existence of a coordinate system for the supergenome $\mathfrak{A}$ is independent of the block order <. However, the order < is crucial for the practical use of the coordinate system.

### Adjacency and betweenness of MSA blocks

The natural starting point for considering adjacency and betweenness of alignment blocks are their constituent intervals $(\mathcal {G},c,i,j,\sigma)$ on a fixed assembly $\mathcal {G}$ and contig *c*. Intervals have a natural partial order defined by $(\mathcal {G},c,i,j,\sigma)\prec (\mathcal {G},c,k,l,\sigma)$ whenever $i<k$ and $j<l$. Two intervals are incomparable in this *interval order* if and only if one is contained in the other. Note that the interval order allows comparable intervals to overlap. We also consider intervals incomparable that belong to different contigs and/or assemblies.

Given three intervals $\alpha =(\mathcal {G},c,i',j',\sigma ')$, $\beta =(\mathcal {G},c,i'',j'',\sigma '')$, and $\gamma =(\mathcal {G},c,i,j,\sigma)$ (on the same genome assembly and contig), we say that $\gamma$ is *between* the two distinct intervals $\alpha$ and $\beta$ if $\alpha \prec \gamma \prec \beta$ or $\beta \prec \gamma \prec \alpha$.

Given a collection $\mathcal {Q}$ of intervals on the same assembly $\mathcal {G}$ and contig *c*, we say that $\alpha =(\mathcal {G},c,i',j',\sigma ')$ and $\beta =(\mathcal {G},c,i'',j'',\sigma '')$ are *adjacent* if there is no interval $\gamma$ between $\alpha$ and $\beta$. We say that $\alpha$ is a *predecessor* of $\beta$ if $\alpha$ and $\beta$ are adjacent and $\alpha \prec \beta$. Analogously, $\alpha$ is a a *successor* of $\beta$ if $\alpha$ and $\beta$ are adjacent and $\beta \prec \alpha$.

#### **Lemma 1**

*Let* $\mathfrak{A}$* be a supergenome and consider the the collection* $\{\pi _{\mathcal {G}}(B)|B\in \mathfrak{A}\}$* of intervals on a given* $\mathcal {G}$.* Then (i) no two intervals overlap, (ii) the interval order* $\prec$ *is a total order on every contig c, (iii) every interval has at most one predecessor and one successor, and hence is adjacent to at most two intervals, and (iv) if* $\gamma$* is adjacent to both* $\alpha$* and* $\beta$,* then* $\gamma$* is between* $\alpha$* and* $\beta$.

#### *Proof*

Property (i) follows directly from the condition. (ii) As a consequence, any two intervals on a fixed contig *c* are comparable, i.e., the restriction of $\prec$ to *c* is a total order. Since only intervals on the same contig can be adjacent, (iii) is an immediate consequence of (ii) and the fact that the number intervals is finite. Property (iv) is now a trivial consequence of the fact that by (iii) $\alpha$ and $\beta$ must be the predecessor and successor of $\gamma$.

A key construction in this contribution is the notion of betweenness relations for alignment blocks.

#### **Definition 3**

Given three blocks $A,B,C \in \mathfrak{A}$, we say that *C* *is between* *A* *and* *B* *with respect to* $\mathcal {G}$ if $\pi _{\mathcal {G}}(C)$ is between $\pi _{\mathcal {G}}(A)$ and $\pi _{\mathcal {G}}(B)$. The ternary relation ${{\mathscr{C}}}{{(\mathfrak{A}}})$ is the defined by $(A,C,B)\in {{\mathscr{C}}}({{\mathfrak{A}}})$ whenever *C* is between *A* and *B* for some assembly ${\mathcal {G}}$.

We note that contradicting betweenness relations resulting from different genome assemblies ${\mathcal {G}}$ are to be expected, i.e., the relation ${{\mathscr{C}}}{{(\mathfrak{A})}}$ will in general not satisfy the properties of a well-formed betweenness relation. We will return to this issue below.

#### **Definition 4**

Two blocks $A,B\in \mathfrak{A}$ are *adjacent* if there is an assembly $\mathcal {G}$ such that $\pi _{G}(A)$ and $\pi _{G}(B)$ are adjacent w.r.t. $\{\pi _{\mathcal {G}}(C)|C\in \mathcal {A}\}$.

It is useful to regard $\mathfrak{A}$ with its adjacency relation as a graph. In order to keep track of the individual contigs, we use an edge-colored multigraph, with $\mathcal {G}$ serving as edge color.

#### **Definition 5**

The *supergenome graph* $\Gamma (\mathfrak{A})$ of an MSA $\mathfrak{A}$ is the directed, edge-colored multigraph whose vertices are the alignment blocks of $\mathfrak{A}$ and whose directed edges (*A*, *B*) connect an alignment block *A* to an alignment block *B* with color $\mathcal {G}$ whenever there are sequence intervals $\alpha \in A$ is a predecessor of $\beta \in B$ in assembly $\mathcal {G}$.

**Fig. 1:**
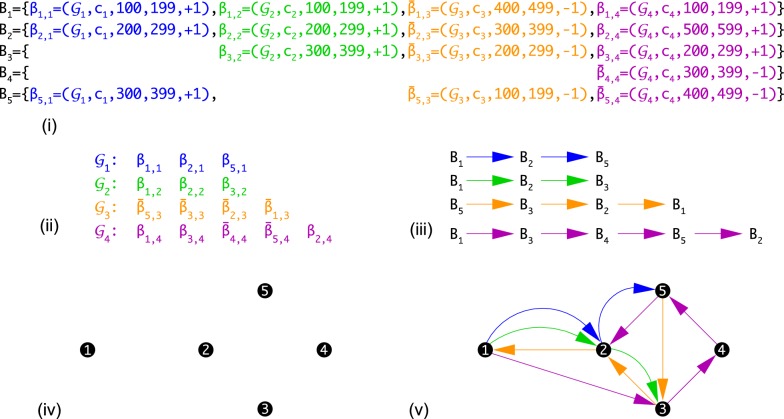
Projections of a supergenome graph. An example how the projection of an artificial MSA $\mathfrak{A}$ to a supergenome graph $\Gamma (\mathfrak{A})$ can be done. Starting point is the MSA $\mathfrak{A}$ shown in **(i)**, which comprises five blocks ($B_1$, ..., $B_5$), each consisting of up to four intervals from the four genome assemblies (distinguished by colors: $\mathcal {G}_1$ blue, $\mathcal {G}_2$ green, $\mathcal {G}_3$ orange, $\mathcal {G}_4$ purple) with one contig each ($c_1$, ..., $c_4$). The intervals are designated as $\beta _{k,l}=(\mathcal {G}_l,c_l,i_{k,l},j_{k,l},+1) \in B_k$ and $\bar{\beta }_{k,l}=(\mathcal {G}_l,c_l,i_{k,l},j_{k,l},-1) \in B_k$.       To construct the supergenome graph first the predecessor of each interval is determined by sorting the intervals separately for each assembly. By assumption, intervals do not overlap. The ordered set of each assembly is shown in panel **(ii)**. Only these orders are relevant in subsequent steps, hence we suppress the positional information from now on. The interval order implies a separate predecessor relation among blocks **(iii)**, with a colored arrow from $B_1$ to $B_2$ implying that $B_1$ is a predecessor of $B_2$ w.r.t. the assembly indicated by the color. The supergenome graph $\Gamma (\mathfrak{A})$ has all blocks of $\mathfrak{A}$ as its vertices **(iv)**. The predecessor relations among the blocks define the colored, directed edges **(v)**

An example how the prejection $\Gamma (\mathfrak{A})$ is done is shown in Fig. 1. The projection of $\Gamma (\mathfrak{A})$ to a constituent assembly $\mathcal {G}$ is a (not necessarily induced) subgraph. As an immediate consequence of Lemma 1, each projection is a disjoint union of directed paths, each of which represents a contig. Conversely, every colored directed multigraph whose restriction to a single color is a set of vertex-disjoint directed paths is a supergenome graph. It therefore makes sense to talk about a supergenome graph $\Gamma$ without explicit reference to an underlying alignment $\mathfrak{A}$.

The structure of the *supergenome graph* strongly depends on the evolutionary history of the genomes that it represents. In the absence of genome rearrangements (i.e., when the only genetic changes are substitutions, insertions (including duplications), and deletions) then all genomes remain colinear with their common ancestor. In other words, a single, canonical [30] global alignment describes a common coordinate system that is unique up to the (arbitrary) order of contigs and each trace [31] of insertions and deletions. In terms of the block adjacency relation, each block has at most two adjacent neighbors in this scenario.

Genome rearrangements are by no means infrequent events [32–35], and thus cannot be neglected. Every breakpoint introduced by a genome rearrangement operation, be it a local reversal or a cut-and-join type dislocation, introduces an ambiguous adjacency, i.e., a block that has two or more predecessors or successors. The task of identifying an appropriate ordering of the MSA blocks therefore is a non-trivial one for realistic data, even in the absence of alignment errors.

### Modeling the “Supergenome Sorting Problem”

Informally, we may consider the “*supergenome sorting problem*” (SSP) as the task of finding an order < (or, equivalently, a permutation $\rho$) of the alignment blocks of $\mathfrak{A}$ *such that the orders of the constituent assemblies are preserved as much as possible*. Somewhat more precisely, we are to find an order < on the vertex set of the supergenome graph $\Gamma (\mathfrak{A})$ that as many of its directed edges as possible are “consistent” with the order <. It is not clear from the outset, however, how “consistency” should be defined for our application. A large number of related models have been proposed and analyzed in the literature that make this condition precise in different ways, leading to different combinatorial optimization problems. We therefore proceed with a brief review of some paradigmatic approaches. A reader primarily interested in our proposed approach to the problem might want to skip this section.

#### Hamiltonian paths

A plausible attempt is to view the SSP as a variant of the Hamiltonian path problem on the *supergenome graph* $\Gamma$. A Hamiltonian path defines a total order of the vertices and therefore a solution to the SSP. This is idea is similar to the use of Hamiltonian graphs for genome assembly from read overlap graphs [36]. There are several quite obvious difficulties, however. First, it is not sufficient to consider only paths that are entirely confined to pass through the adjacencies. The simplest counterexample consists of only 4 MSA blocks $B_1$, $B_2$, $B_3$ ,$B_4$ and three assemblies $\mathcal {G}_1$, $\mathcal {G}_2$, $\mathcal {G}_3$. Given eight sequence intervals $\beta _{k,l}=(\mathcal {G}_l,c_{k,l},i_{k,l},j_{k,l},+1) \in B_k$ we construct the following alignment:

$$\begin{aligned} \begin{array}{*{20}{c}} {}&{}{{B_1}}&{}{{B_2}}&{}{{B_3}}&{}{{B_4}}\\ {{g_1} = }&{}{{\beta _{1,1}}}&{}{}&{}{}&{}{{\beta _{4,1}}}\\ {{g_2} = }&{}{{\beta _{1,2}}}&{}{{\beta _{2,2}}}&{}{}&{}{{\beta _{4,2}}}\\ {{g_3} = }&{}{{\beta _{1,3}}}&{}{}&{}{{\beta _{3,3}}}&{}{{\beta _{4,3}}} \end{array} \end{aligned}$$

This situation arises in practice e.g. when $B_2$ and $B_3$ are two independent, unrelated inserts between $B_1$ and $B_4$. The block adjacency graph is the graph

$$\begin{aligned} {B_2-B_1-B_4-B_3,} \end{aligned}$$

which violates the desired betweenness relation $(\beta _{1,3},\beta _{3,3},\beta _{4,3})$.

In this case there are only two biologically correct solutions: $B_1<B_2<B_3<B_4$ (or the inverse order) and $B_1<B_3<B_2<B_4$ (or its inverse). In either case, the solution contains two consecutive blocks ($B_2$ and $B_3$) that are not adjacent in the block graph. This example also serves to demonstrate that the block graph alone does not contain the complete information on the supergenome. It appears that in addition we will need to know the *betweenness* relation among the blocks i.e., that both $B_2$ and $B_3$ are between $B_1$ and $B_4$.

#### Feedback arc sets and topological sorting

An other possibility to determine a well-defined order of the vertices of the supergenome graph $\Gamma$ is to first extract a maximum acyclic subgraph and then to compute a topological sorting of this subgraph. An equivalent formulation asks for the removal of a minimum set of edges that close cycles. This Maximum Acyclic Subgraph or Minimum Feedback Arc Set problem (MFAS) is well-known to be NP-hard [37]. Nevertheless fast, practicable heuristics have been devised, see e.g. [38, 39]. From the resulting directed acyclic graph (DAG) an admissible ordering of blocks can be obtained efficiently by topological sorting [40]. A closely related approach is the Linear Ordering Problem (LOP): given a complete weighted directed graph, find a tournament with maximum total edge weights [41]. It yields essentially the same model since LOP and MFAS can be transformed into each other quite easily [42]. Cost functions designed to define consensus orderings for sets of total and partial orders have been considered in different fields starting with the work of Spearman [43] and Kendall [44], see also e.g. [45–47]. The reconciliation of partial orders in investigated in detail e.g. in [48].

**Fig. 2:**
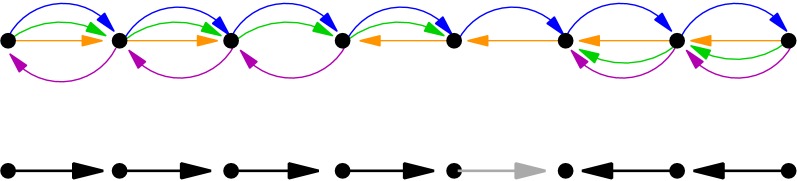
Minimum feedback arc set (MFAS) does not necessarily yield an optimial solution of the SSP. Due to the arbitrariness of the orientation of the edges, the best solution of the SSP may contain cycles, which by definition is excluded in MFAS. Top: *supergenome graph* representation $\Gamma (\mathfrak{A})$ of an artificial alignment $\mathfrak{A}$. Bottom: simplified solution of the (uniformly weighted) MFAS. To turn $\Gamma (\mathfrak{A})$ into an acyclic graph, at least one edge has to be deleted. Two such solutions exist, differing only by the orientation of the gray arrow. The corresponding topological sorting breaks the genome into two distinct colinear pieces with opposite orientation. There is, however, a consistent order of the entire graph—the linear left-to-right or right-to-left order is consistent

The key problem of modeling the SSP in terms of MFAS is highlighted in Fig. 2. It shows that even when undirected adjacencies would allow for a perfect solution, it may not be uncovered directly by the MFAS approach.

#### Simultaneous consecutive ones and matrix banding

Instead of adjacencies we may consider the incidence matrix $\mathbf {A}$ of the supergenome graph $\Gamma$ and try to sort both the alignment blocks and their adjacencies in such a way that, to the extent that this is possible, (i) adjacent blocks are consecutive and (ii) adjacencies that have a block in common are consecutive. In more formal terms, we wish to sort both the rows (alignment blocks) and columns (adjacencies) of the incidence matrix in such a way that rows and columns show all non-zero entries consecutively. A rectangular matrix $\mathbf {A}$ that admits such a pair of row and column permutations is said to have the *simultaneous consecutive ones property* (C1S) [49]. This is possible if $\Gamma$ is a union of paths. Note that instead of adjacencies we could also cover the graph with short paths $\wp _k$. In this case column *k* identifies the vertices incident with path *k*. Again, if $\Gamma$ is a union of paths, the path-incidence matrix satisfies (C1S). It is not difficult to see [49] that $\mathbf {A}$ satisfies (C1S) if and only if $\mathbf {A}$ has the well-studied consecutive ones property [50, 51] for both its rows and columns. Thus (C1S) can be checked in linear time [50]. Furthermore, Tucker’s characterization of (C1S) in terms of forbidden sub-matrices [52] also carries over. Some direct connection between the consecutive ones property and the Betweenness Problem, which we will consider below, are discussed in [53].

In general, $\mathbf {A}$ will now have the consecutive ones property. The problem of identifying a minimal number of columns (adjacencies) whose removal leaves a (C1S) matrix is NP-complete [49]. In practise it may be desirable to quantify the extent of the violation of (C1S) in terms of intervals of consecutive zeros enclosed by the two 1s. For instance, one may want to use $\omega =\sum _i h(\ell _i)$, where the sum runs over all intervals *i* of consecutive zeros enclosed by the two 1s, and $h(\ell _i)\ge 0$ is some contribution that monotonically grows with the length $\ell _i$ of the 0-interval. For a given ordering of the rows and columns, the total violation is quantified as the sum of the $\omega$ values. It should be noted, however, that (C1S) does not imply $\Gamma$ is a union of disjoint paths, i.e., that $\Gamma$ is a valid supergenome graph.

A related set of optimization problems is concerned with reducing the bandwidth of matrices, i.e., the maximal distance of non-zero entries from the diagonal (in a symmetric case) or the parameter $\min (l,u)+l+u$ (for rectangular matrices); here $u=\max _{\mathbf {A}_{ij}\ne 0} (i-j)$ and $l=\max _{\mathbf {A}_{ij}\ne 0} (j-i)$ [54]. In the symmetric case, several good heuristics are known, starting with the Cuthill-McKee [55] and GPS [56] algorithms even though the problem is NP-hard [57], while the general case has received much less attention [54]. Bandwidth reduction methods do not eliminate “bad” adjacencies that eventually determine bandwidth. The resulting ordering of rows and columns thus may be very inaccurate locally.

#### Bidirected graphs

**Fig. 3:**
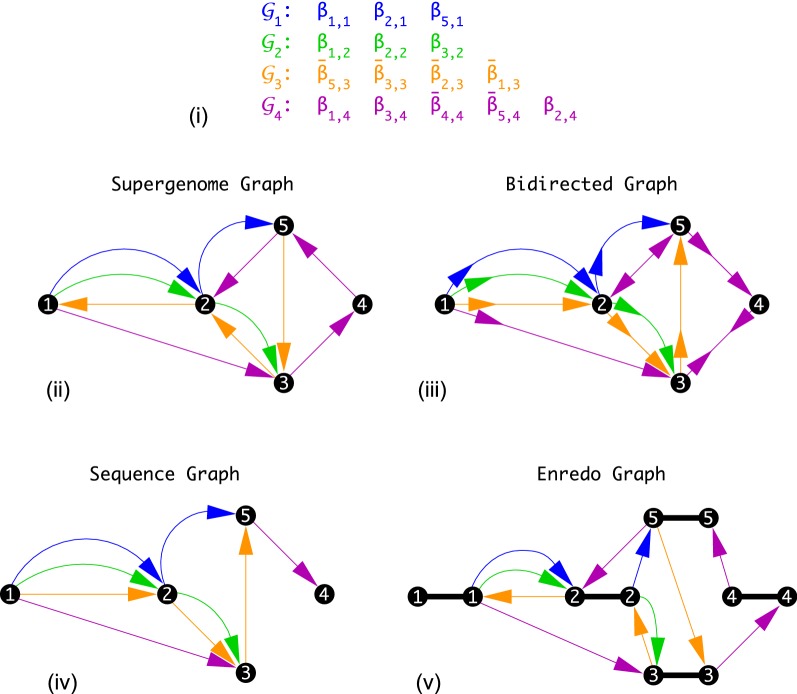
Comparison of different graph definitions. Consider an alignment comprising of five MSA blocks ($B_1$, $B_2$, $B_3$, $B_4$, $B_5$) in four assemblies (indicated by different edge colors: $\mathcal {G}_1$ blue, $\mathcal {G}_2$ green, $\mathcal {G}_3$ orange, $\mathcal {G}_4$ purple), consisting of a total of sequence intervals denoted by $\beta _{k,l}=(\mathcal {G}_l,c_{k,l},i_{k,l},j_{k,l},+1) \in B_k$ and $\bar{\beta }_{k,l}=(\mathcal {G}_l,c_{k,l},i_{k,l},j_{k,l},-1) \in B_k$ for positive and negative orientation, respectively. In the drawings, block $B_i$ is simply denoted by *i*. Most of the edges in the different graphs represent the adjacency of two intervals in one of the constituent assemblies. These edges are displayed in the corresponding color. **(i)** Genome order and orientation of the intervals. Every line shows one assembly and the order on this assembly. **(ii)** The supergenome graph. **(iii)** The bidirected graph. Note that the edges are the same as in the supergenome graph except for the shown direction. **(iv)** The sequence graph of Haussler et al. [29]. Note that the edges (3,4) and (5,2) are not part of the graph because of the change in orientation. **(v)** The enredo graph. An example of the other special graph structures discussed in Kehr *et al.* [58]

Several specialized graph structures have been introduced recently to tackle the problem of ordering sequence blocks, which is a problem very similar to SSP. Among these constructions are A-Bruijn graphs, Enredo graphs, and Cactus graphs, see [58] for a review. A key insight of [58] is that these graph representations are equivalent in the sense that they can be transformed into each other. They differ, however, in additional information extracted from the input alignment that is stored as vertex and edge labels, see Fig. 3. For instance, the bidirected graphs of [28] have the same underlying graph as our supergenome graph. While the supergenome graph used a standard directed graph structure, bidirected graphs encode directional information independently for the endpoint of each edge, distinguishing three cases: (i) adjacent alignment blocks have the same orientation, (ii) the connected blocks switch from minus to plus orientation, or (iii) vice versa. The latter two cases indicate a change of orientation between two changes. In [28], this basic structure is extended by additional transitive edges given by a legal path through the graph with an exponential weight function. The task is then to find a consistent (non-conflicting) set of edges with maximal weight. This form of the weight function takes into account that, due to genetic linkage, the more closely positioned on a genome two blocks are, the less likely it is that the edge between these blocks is broken. This gives higher weight to locally correct block positions and orientations.

While this sorting problem is (NP-) hard in general, the sets of genomes considered by the authors are particularly suitable for this kind of calculations. The genomes considered for pangenome construction are typically closely related, or even of the same species. Thus one can expect many paths with high weights of the conserved consensus [29]. This approach also fits well to the analysis of genomic regions that are under constraint to maintain syntenic order for functional reasons, such as the MHC locus used as an example in [28]. In distantly related genomes, however, synteny tends not to be well preserved. In addition, we are interested in particular in data sets that contain genomes in preliminary draft forms, i.e., linkage information that is at least partially limited to short contigs or scaffolds. As a consequence we have less confidence in linkage and orientation information than one can expect in a typical pangenome scenario.

#### Sequence graphs

The sequence graphs of Haussler et al. [29] are also closely related to the *supergenome graph* representation $\Gamma (\mathfrak{A})$ of a multiple alignment $\mathfrak{A}$. The key difference is that the the orientation of the blocks is used to determine the direction of the edge. Two adjacent intervals with negative orientation thus imply an edge that is reversed compared to the supergenome graph. This situation is problematic in the case where the orientation of the intervals is switched. In [29] a preprocessing step is performed to minimize the number of such edges. The sequence graph approach was designed for the comparison of human genomes of different individuals. In such a scenario, the resulting information loss is small and does not present a practical problem. It is likely to become an issue, however, for large phylogenetic distances with frequent genome rearrangements.

The natural formulation of SSP on a sequence graph is to find a vertex ordering that minimizes weighted feedback edges and the average cut width. These optimization criteria ensure that the successor relations are mostly kept intact and at the same time successors are placed close to each other in the solution. Both problems the Minimum Feedback Arc Set Problem [37] and the Average Cut-Width Minimization Problem [59–61] are known to be NP-hard. Cutwidth minimization problems ask for a linear ordering of the vertices of a graph such that the average or maximum number of edges spanning across the gap between a pair of consecutive vertices is minimized. Conceptually, cutwidth problems are quite similar to bandwidth problems [62]. In [29] a heuristic is presented that first extracts a totally ordered “backbone” and then inserts the remaining vertices into the backbone order that is kept intact in the process. While the presence of a global common backbone order is a well founded assumption for pangenomes of a single or very closely related species, it is violated substantially for phylogenetically diverse data.

### Betweenness problems

In this work we interpret SSP as a betweenness (ordering) problem rather than a vertex ordering problem on a directed graph. Instead of (oriented) adjacencies, which are defined on pairs of blocks, one considers the relative order of three blocks:

Betweenness Problem [63, 64]: *Given a finite set* *X* *and a collection* ${{\mathscr{C}}}$ *of triples from* *X*, *is there a total order on* *X* *such that* $\forall (i,j,k)\in {{\mathscr{C}}}$ *either* $i<j<k$ or $i>j>k$?

This decision problem is NP-complete [63, 64]. A triple $(i,j,k)\in {{\mathscr{C}}}$ is called a betweenness triple.

The Betweenness Problem can be adapted to model the SSP by means of a suitable cost function *b* designed to penalize violations of the betweenness relation. Consider an order $\rho$ used to coordinatize the supergenome. We may think of $\rho$ as a bijective function from $[1,\ldots ,|\mathfrak{A}|]\rightarrow \mathfrak{A}$. In other words, we number the blocks contained in $\mathfrak{A}$ and work on this set of block indices.

For $i<j<k$ we set $b_{\rho ,{\mathcal {G}}}(i,j,k)=1$ if the projections of the three alignment blocks $\rho (i)$, $\rho (j)$, and $\rho (k)$ exist and violate the betweenness relation for a given assembly ${\mathcal {G}}$, i.e., if $\pi _{\mathcal {G}}(\rho (i))$, $\pi _{\mathcal {G}}(\rho (j))$ and $\pi _{\mathcal {G}}(\rho (k))$ are located on the same contig and $\pi _{\mathcal {G}}(\rho (j))$ is not located between $\pi _{\mathcal {G}}(\rho (i))$ and $\pi _{\mathcal {G}}(\rho (k))$. Otherwise we set $b_{\rho ,{\mathcal {G}}}(i,j,k)=0$. A natural cost function is now the total number of betweenness violations

1 $$\begin{aligned} b(\rho) := \sum _{\mathcal {G}\in G(\mathfrak{A})} \sum _{i<j<k} b_{\rho ,\mathcal {G}}(i,j,k)\,, \end{aligned}$$

where $G({\mathfrak{A}})$ is the set of genome assemblies that are contained in $\mathfrak{A}$. If genome evolution were to preserve gene order, i.e., only local duplications and deletions are allowed, the betweenness relation of the ancestral state would be preserved, guaranteeing a perfect solution $\rho$ with $b(\rho)=0$.

Since this decision problem is NP-complete [63, 64], so is the problem of optimizing $b(\rho)$ NP-hard. The proof is shown in Additional file 1: Section 1 The cost function $b(\rho)$ involves the sum over all triples of alignment blocks and thus is fairly expensive to evaluate. It is interesting in practice, therefore, to consider a modified cost function that restricts the sum in Eq. (1) to local information. This idea leads us to the rather natural extension of the Betweenness Problem to colored multigraphs.

#### **Definition 6**

Given an (undirected) *colored multigraph* $\hat{\Gamma }=(V,E)$, the triple (*i*, *j*, *k*) is part of the collection ${{\mathscr{C}}}(\hat{\Gamma })$, iff there are edges $\{i,j\} \in E$ and $\{j,k\} \in E$ with color $\mathcal {G}$.

Colored Multigraph Betweenness Decision Problem: given the colored multigraph $\hat{\Gamma }=(V,E)$, is there a total order on *V* such that $\forall (i,j,k)\in {{\mathscr{C}}}(\hat{\Gamma })$ either $i<j<k$ or $i>j>k$?

The reformulation as an optimization problem that maximizes the number of edges is straightforward: Colored Multigraph Betweenness Problem: Given a colored multigraph $\hat{\Gamma }=(V,E)$, find a total order on *V* such that $E^* \subseteq E$ is maximal under the condition that $\forall (i,j,k)\in {{\mathscr{C}}}(V,E^*)$ either $i<j<k$ or $i>j>k$.

This problem can be viewed as an analog of the Minimum Feedback Arc Set problem [38] for betweenness data. To our knowledge is has not been studied so far.

#### **Lemma 2**

*The (decision version of the)* *Colored Multigraph Betweenness Problem is NP-complete*.

#### *Proof*

Every set ${{\mathscr{C}}}(\hat{\Gamma })$ of triples can be obtained from an edge-colored multigraph $\hat{\Gamma }$ (with vertices corresponding to alignment blocks and colored edges corresponding to adjacencies deriving from a genome identified by the color). Thus, the total order on the vertices of $\hat{\Gamma }$ is a solution of the Colored Multigraph Betweenness Problem if and only if the answer to the NP-complete Betweenness Problem is positive. In Additional file 1: Section 1 the reduction in both directions is shown.

In the example of Fig. 2 the optimal solution of the Colored Multigraph Betweenness Problem retains all *unordered* adjacencies and creates a unique coordinatization (up to orientation) that leaves all alignment blocks ordered as drawn.

#### Seriation

An alternative framework for solving the SSP by construction of a preferred ordering is seriation. The Robinson seriation problem [65] starts from a dissimilarity measure $d:X\times X\rightarrow \mathbb {R}$, and seeks a linear order $\rho$ on *X* that satisfies the inequality

2 $$\begin{aligned} \max \{d(\rho (i),\rho (j)),d(\rho (j),\rho (k))\}\le d(\rho (i),\rho (k))\,. \end{aligned}$$

A dissimilarity *d* for which an ordering $\rho$ exists that satisfies Eq. (2) for all $\rho (i)<\rho (j)<\rho (k)$ is called *Robinsonian*. It is worth noting that Robinsonian dissimilarities are intimately related with pyramidal clustering problems [66, 67].

The seriation problem [65, 68] consists of finding a total order for which the given pairwise distances violates the Robinson conditions as little as possible. To link this seriation problem with the Colored Multigraph Betweenness Problem or Betweenness Problem we consider a collections ${{\mathscr{C}}}$ of triples such that

3 $$\begin{aligned} (\rho (i),\rho (j),\rho (k))\in {{\mathscr{C}}} \text { implies} \max \{d(\rho (i),\rho (j)),d(\rho (j),\rho (k))\}< d(\rho (i),\rho (k)) \end{aligned}$$

Clearly, if the dissimilarity is Robinsonian, then $\rho$ defines a total order on *X* that solves the Betweenness Problem for $(X,{{\mathscr{C}}})$.

The relevant optimization task in our context is therefore to minimize the number of ordered triples that violate Eq. (2). A variety of heuristics for this problem have been developed, see e.g. [69]. It is important to note, however, that in our setting the distance between alignment blocks is not defined directly. In order to obtain a seriation problem that approximates the Supergenome Sorting Problem we will have to resort to a heuristic that summarizes the distances between two blocks in all genomes and reflects the betweenness relationships. For pangenome-like models, the cost function advocated in [28] is a very plausible choice.

### Graph simplification

Each of the plausible models for the “Supergenome Sorting Problem” discussed in the previous sections leads to NP-hard computational problems. The size of typical genome-wide alignments by far exceeds the range where exact solutions can be hoped for, except possibly for the smallest and most benign examples such as the ones used as examples in [17]. We therefore will have to resort to fast heuristics. In this section we focus on the conceptual ideas behind the simplification steps. More detailed implementation details are given in the "Methods" section.

Nevertheless it is possible to isolate certain sub-problems that can be solved exactly and independently of the remainder of the input graph. Since “linearized” portions of the vertex set can be contracted to a single vertex set, this leads to a reduction of problem size.

#### **Lemma 3**

*If the supergenome graph* $\Gamma$* is a directed acyclic graph then topological sorting of* $\Gamma$* solves the* *Colored Multigraph Betweenness Problem*.

#### *Proof*

In this case betweenness is established exactly by the directed paths in the DAG. Hence any topological sorting preserves all betweenness triples of *G* and thus presents a perfect solution to the Colored Multigraph Betweenness Problem as well.

This simple observation suggests to identify subgraphs with DAG structure and to replace them with a representative for each replaced DAG. These can later be replaced by the solution that is created with topological sorting. We note that this does not necessarily preserve optimality. It is conceivable that a local DAG structure has to be broken up into two disjoint subsets that are integrated in larger surrounding structures in a way that requires reversal of the edge directions in one or even both parts. Nevertheless, if the local DAG structures are sufficiently isolated they are likely to be part of the optimal solution as a unit. The motif that describes such a local DAG structure has been introduced as “superbubble” in the context of graph structures arising in sequence assembly problems [70, 71].

**Fig. 4:**
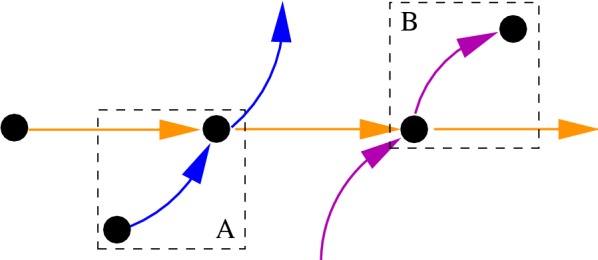
A Sink and Source Example are shown here. In this part of a bigger supergenome graph a sink and a source exists. The source is in field A. It has only one successor and thus can be merged with this successor. The sink is in field B. It has only one predecessor and thus can be merged with this predecessor

Source and sink vertices *s* in the supergenome graph with only a single neighbor *t* are conceptually a special case of superbubbles. These can be sorted together with their unique neighbor *t*. $\Gamma$ is thus simplified by contracting *s* and *t*, i.e., placing the source *s* immediately before *t* and sink *s* immediate after *t*. An example of such a source and a sink can be seen in Fig. 4.

In some cases it is helpful to reverse the direction of the coordinate system of a single species. This is in particular the case when a single genome is reversed compared to all others. The inversion of an entire path does not change the solution of the Colored Multigraph Betweenness Problem but can make it easier to apply some of the reduction heuristics discussed above. In particular, if the relative orientation of the coordinatizations could be fixed in an optimal manner, the betweenness problem reduces to a much easier topological sorting problem. Finding this optimum, however, is equivalent to the Betweenness Problem, which is a NP-hard. Hence, we again have to resort to local heuristics.

#### **Definition 7**

Let $\Gamma = (V,E)$ be a supergenome graph. A pair of vertices $v,w\in V$ such that there are edges (*v*, *w*) and (*w*, *v*) in *E* is a *mini cycle*.

Mini-cycles are naturally removed by removing one of the two edge directions between *v* and *w*. More precisely, the less supported direction of an edge is dropped. The estimate for support is evaluated in a region around a mini-cycle since adjacent mini-cycles may yield contradictory majority votes.

#### **Definition 8**

Two mini-cycles are connected with each other if they share a vertex. A mini-cycle complex $\mathfrak{C}$ is a maximal connected set of mini-cycles.

#### **Lemma 4**

*The mini-cycle complexes of a supergenome graph* $\Gamma$ *form a unique partition of the set of all minicycles. Any two classes of this partition are vertex and edge disjoint.*

#### *Proof*

Consider the the graph *H* whose vertices are the mini-cycles, and there are edges between any two mini-cycles that share at least one vertex. Then every mini-cycle complex $\mathfrak{C}$ is a connected component of *H*. Since the connected components of a graph are uniquely defined, disjoint, and form a cover, they partition the vertex set of *H*. Every minicycle, furthermore, forms a connected subgraph of $\Gamma$ by construction. Since any two minicycles that contain a common vertex belong to the same mini-cycle complex, two mini-cycle complexes cannot have a vertex in common. This implies that they are also edge disjoint.

The mini-cycle complexes therefore can be resolved independently of each other. The target is to remove edges that create cycles in order to obtain a DAG that can then be subjected to topological sorting. However, this topological sorting is a solution of the Colored Multigraph Betweenness Problem for a subgraph. This is still a hard problem, so that we again utilize a heuristic approach. In this step we only attempt to remove mini-cycles. Cycles that connect mini-cycle complexes with each other or with other vertices in the graph are therefore left untouched and have to be dealt with in a subsequent step.

**Fig. 5:**
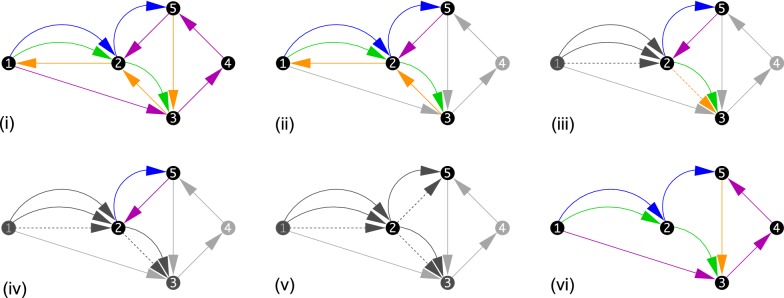
Step-wise resolution of a complex of mini-cylces. **(i)** Starting point. **(ii)** The mini-cycle complex is highlighted. The complex is created from the mini-cylces $\{1,2\}$, $\{2,3\}$, and $\{2,5\}$. Note that the edges (1, 3) and (5, 3) are not contained in the complex. The best supported directions are between (1, 2). **(iii)** This direction between (1, 2) is then set. The orange edges are therefore reversed (marked by dashed lines). The adjacency $\{1,2\}$ is decided and is no longer considered (marked with dark grey). **(iv)** In this step the best supported direction is (2, 3) and the graph is updated correspondingly. **(v)** Adjacency $\{2,5\}$ is left. No direction is superior. Since vertex 2 was solved for previously it is now used. This leads to direction (2, 5). **(vi)** Then the completed complex is decided and the edges that are contradictionary with the decisions are removed. Note that the circle 3, 4, 5, 3 that was not part of the complex is not removed

The local sorting within a complex $\mathfrak{C}$ is achieved by considering adjacencies. To this end we annotate each adjacency with the number of edges and the ratio of the edges in the two directions. We identify the best supported edges as those with a high multiplicity and a strong bias for one direction over the other. This choice of a direction is then propagated. If a directed edge has more than one possible successor, we first propagate along the one with the largest support for the proposed direction. The issue now is when exactly to stop propagating this information. Clearly, it is forbidden to orient an edge that would close a directed cycle. Any such edge is instead seeded with the reverse directional information.

As part of this procedure it is possible that parts of a directed path from a given genome received contradictory orientations in different regions. If this is the case, the edge crossing the boundary between the differently oriented regions must be removed. Finally, the heuristic may terminate and still leave some edges unoriented. This indicates that the orientations are contradictory and need to be reversed. An example of the mini-cycle resolution process is shown in Fig. 5.

## Methods

### Curation of input data sets

We investigate here three genome-wide data sets. The smallest set, referred to as $\mathbf {B}$ (bacteria) below, is an alignment of four *Salmonella enterica* serovars. This alignment was produced with Cactus [27] using the *Salmonella enterica Newport* genome as reference and comprises 13, 416 blocks, 50, 932 sequence fragments, and 18, 047, 456 nucleotides. The medium-size set, termed $\mathbf {Y}$ (yeast), is an alignment of seven yeast species that uses the *Saccharomyces cerevisiae* genome as references. It comprises 49, 795 alignment blocks composed of 275, 484 sequences fragments that contains 71, 517, 259 nucleotides. The third, much larger set $\mathbf {F}$ (fly) is a alignment of 27 insect species that uses the *Drosophila melanogaster* genome as references. It comprises 2, 112, 962 blocks composed of 36, 139, 620 sequence fragments hat contains 2, 172, 959, 429 nucleotides. For more detailed information of the data sets refer to Additional file 1: Section 2.

The two large genome-wide multiple sequence alignments were produced by the multiz pipeline and were downloaded from the UCSC genome browser [72]. They are, as discussed above, injective but not irredundant. In order to remove spurious alignment blocks we filter the input blocks with respect to first length, then score, and finally mutual overlap. Very short alignment blocks are almost certainly either spurious matches or they were inserted to bridge gaps between larger blocks. Consequently, they convey little or no useful information for our purposes. We therefore remove all blocks with a length $\le 10$ nt.

Since genome-wide alignments tend to contain also very poorly aligned regions we require a minimum similarity, expressed here in the form of sum-of-pairs blastz scores [73]. Since these scale linearly with the number of columns $\ell (B)$ of the alignment block *B* and the number $$\left( {\begin{array}{c}r\\ 2\end{array}}\right)$$ of pairwise alignments formed by the *r* rows in *B*, we normalize with $$\left( {\begin{array}{c}r\\ 2\end{array}}\right) \ell (B)$$ to obtain a similarity measure that is independent of the size of the alignment block. Based on the parametrization of blastz, we set the threshold at a normalized score of $-\;30$, which corresponds to the gap extension penalty.

The coordinatization of the supergenome depends on the uniqueness of coordinate projections. There are three major reasons to observe overlaps, i.e., genomic regions that appear in more than one alignment: (i) the sequence is duplicated in some species. Then multiz tends to align the corresponding unduplicated sequence to both duplicates. (ii) Spurious similarities in particular in poorly conserved regions may lead to alignments containing a sequence element twice at the expense of the second copy. (iii) Short overlaps at the end of blocks may appear due to difficulties in determining the exact ends of alignable regions. The first two causes introduce undesirable noise and uncertainties. Therefore, we remove all such overlapping blocks in which sequences from the species overlap. Since there is no easy way to determine which one of two overlapping blocks is likely correct, we opt to remove both copies. The third case, in contrast, does not disturb the relative order of alignment blocks and thus can be ignored. The overlap filter is applied after low quality alignments already have been removed from the data set.

We tolerate an overlap of 20 nt at the borders of alignment blocks. This cutoff is designed to remove ambiguous alignments, while avoiding the removal of alignment blocks that overlap by a few nucleotides owing to overlapping extensions of local blastz seeds. In addition we remove sequences that completely overlap other sequences regardless of their size to further reduce the noise introduced by spurious alignments. We opt here for a stringent procedure and remove all alignment blocks that contain sequences tagged for removal. In practice, this step removes only a tiny fraction of the blocks and thus does not significantly influence the coverage of the retained data.

The initial data filtering steps removed almost $35\%$ ($40\%$, $30\%$) of the blocks from data set $\mathbf {F}$, ($\mathbf {Y}$, $\mathbf {B}$). The majority were eliminated because of their minimal length. About $8.5\%$ ($27\%$, $0\%$) of the blocks were removed because they contained non-unique sequences. The sequences in the blocks that are removed with all filters contain less then $15\%$ ($26\%$, $0.4\%$) of the nucleotides in the alignment. Hence more than $85\%$ ($74\%$, $99\%$) of the sequence information of the alignment is intact and the quality of the data is significant better. A more detailed summary of the filtering is compiled in Additional file 1: Section 2.

### Graph simplification and DAG construction

**Fig. 6:**
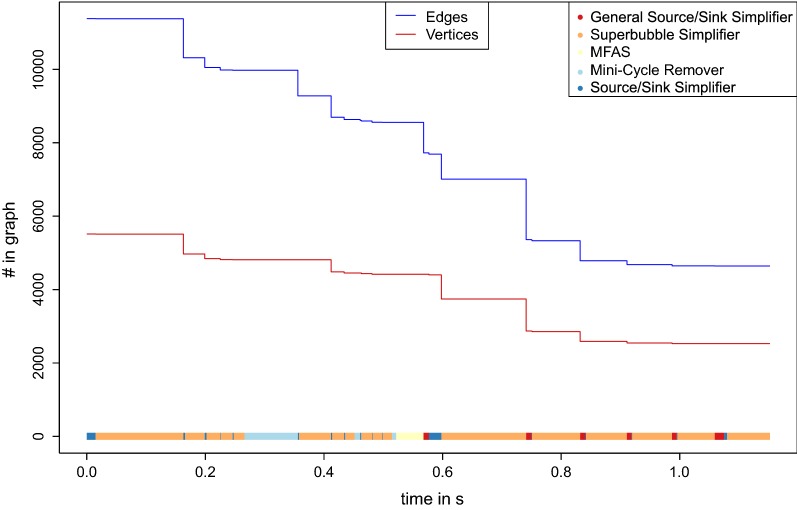
Simplification process for data set $\mathbf {B}$. The size of the graph (number of edges and vertices in the graph) is shown while the simplifier and the MFAS are applied. The running time is computed on a Intel(R) Xeon(R) CPU E7-8860 processor with 32 Gb RAM. At the bottom, the different processes are shown as color-coded bars

**Fig. 7:**
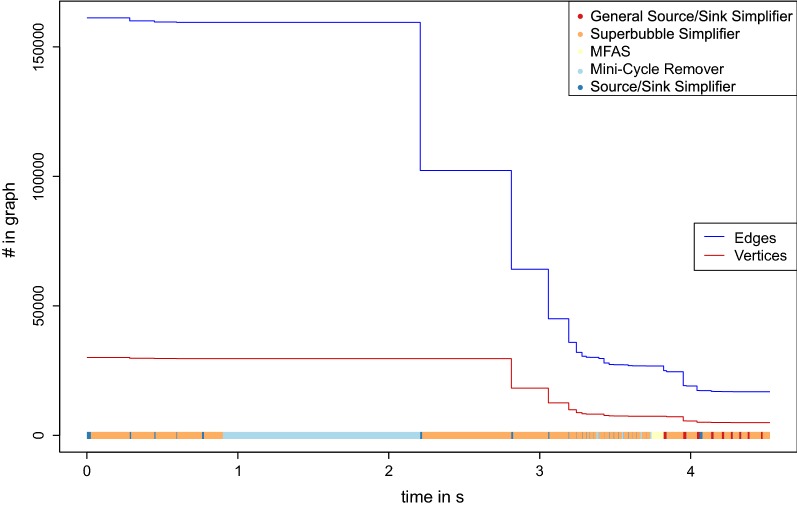
Simplification process for data set $\mathbf {Y}$. The size of the graph (number of edges and vertices in the graph) is shown while the simplifier is applied and the MFAS are solved. The running time is computed on a Intel(R) Xeon(R) CPU E7-8860 processor with 32 Gb RAM. At the bottom, the different processes are shown as color-coded bars.

**Fig. 8:**
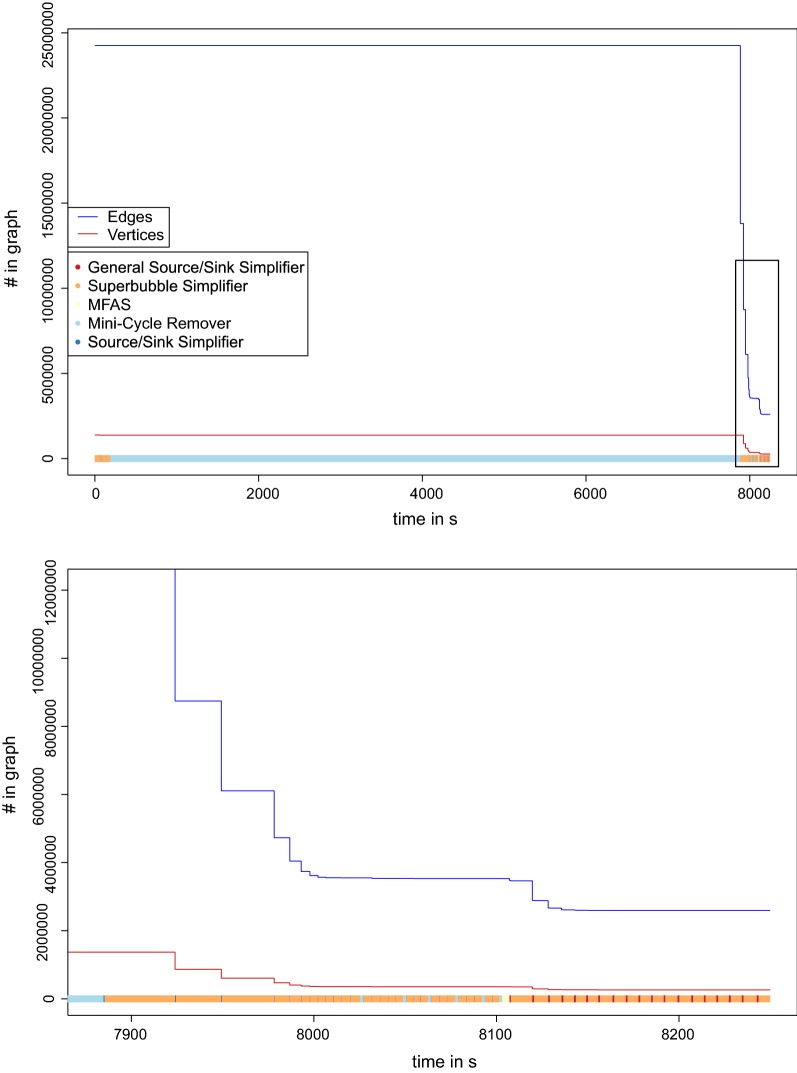
Simplification process for data set $\mathbf {F}$. The size of the graph (number of edges and vertices in the graph) is shown while the simplifier is applied and the MFAS is solved. The running time is computed on a Intel(R) Xeon(R) CPU E7-8860 processor with 32gb ram. At the bottom, the different processes are shown as color-coded bars

The algorithmic ideas and their justifications for the graph reduction steps have already been discussed in "Theory" section. Here we briefly address implementation issues as well as particular choices of cost functions and parameters that were discussed in a more general setting above.

The filtered data is used to create an initial supergenome graph. Then we iterate the three different graph simplifiers until no further reduction steps can be applied: the mini-cycle remover, the source/sink simplifier, and the superbubble simplifier. The individual simplifiers are straightforward implementations of the basic ideas outlined above. The mini-cycle remover first identifies the mini-cycles, aggregates them into non-overlapping complexes, and then proceeds to remove contradictory edges in a greedy manner. The other two simplifiers first check for each vertex in the input graph whether it is a valid sink, source, or starting vertex of a superbubble. Pseudocode of the simplifiers is given in Additional file 1: Section 4.

The mini-cycle remover works more effectively on a single big complex than on many small ones separated by narrow gaps. The other two simplifiers therefore are applied until a fixed point is reached to close some of these gaps. The entire procedure is then iterated until the minicycle remover cannot change the graph any further.

Once a fixed point is reached we attempt to remove directed cycles. This amounts to solving the Minimum Feedback Arc Set Problem, which is known to be NP-hard [37]. Given the size of our input graphs we have to resort to linear-time heuristics. We use Algorithm GR [38] because it is known to work particularly well on sparse graphs. Cycle removal typically creates new possibilities to simplify the graph. For instance, a sink is created whenever the last outgoing edge of a vertex is removed. The new sink can then be simplified further. The graph simplifiers are therefore applied again after the cycle removal step.

The minicycle remover is not used in this second pass because it is not applicable to DAGs by construction. Instead, we use a generalized version of the source/sink simplifier in which a source *s* may have more than a single successor *v*, provided *v* is a predecessor of all other successors of *s*. The position of source *s* in the DAG is determined by *v* and thus *s* can be placed immediately before *v*. The corresponding arrangements for a sink and its predecessor is treated analogously.

The running time and the minimization of the data set while this process is applied is shown in Figs. 6, 7 and 8.

### Seriation

Finally, the common coordinate system is created by seriation of the DAG. The resulting supergenome, i.e. linear order of the vertices of the graphs corresponds to a linear order of all blocks. In particular, vertices resulting from a simplifier may contain more than one block. Those blocks, however, are already sorted and thus are inserted as a single block. Seriation is naturally divided into two steps. First, topological sorting is used to calculate an initial linear ordering from the DAG. Kahn’s algorithm [40] is a classical solution to the topological sorting problem. For our purposes it is desirable that, if possible, two nodes *v* and *w* are placed consecutively whenever there is an edge (*v*, *w*) in the final DAG. To this end we modify Kahn’s algorithm by sorting the successors of a node in the order of evidence for their adjacency in the original data.

The order obtained in this manner may not be optimal w.r.t. its agreement with the order of the blocks in the genomes. It provides a good starting point, however, for the final optimization step, which we phrase as minimizing the number $\tau$ of triplets (*i*, *j*, *k*) for which the Robinson condition, Eq. (2), page 12, is violated. We use the distance measure

4 $$\begin{aligned} d(i,k)= {\left\{ \begin{array}{ll} \frac{1}{|(i,k)|} &{}\quad \text {if an edge }(i,k) \text { exists,}\\ \min _{i<j<k}\{d(i,j)+d(j,k)\} &{}\quad \text {if a path from { i} to { k} through { j} exists,}\\ \infty &{}\quad \text {if no path from { i} to { k} exists,} \end{array}\right. } \end{aligned}$$

where |(*i*, *k*)| is the number of edges from *i* to *k*. Since *d* is a good measure of co-linearity only for short distances, we limit the path length in Eq. 4 to a small number of *l* edges. We set $l=10$ in our implementation. In addition this reduces the effort of computing the distances from $O(|V|^2)$ to *O*(|*V*|) as a consequence of the sparsity of the input graph.

We use a gradient descent-like optimization algorithm to minimize $\tau$. We say that two nodes are siblings if they either share a predecessor in the DAG or if they are both sources. The move set for the gradient descent consists of swaps of siblings only. In addition, we allow to move a node directly in front of its sibling. The discrete gradient is computed exhaustively by generating and evaluating each potential move. Since non-overlapping swaps do not influence each other, we greedily execute a maximal set of non-overlapping swaps in a single optimization step.

### Assessment of the quality of supergenome coordinate systems

Since no ground truth is available for this problem and the construction of simulated benchmarks for genome wide multiple sequence alignments would be a research project in its own right, we have to resort to measuring quantities that are informative about the final choice of the coordinate system.

A straightforward measure is the distribution of distances in the output coordinate system of alignment blocks that are contiguous in at least one input genome. Since we are not interested in the length of alignment blocks, distance is measured here not in terms of sequence length but in terms of the number of alignment blocks, so that adjacent blocks have distance 0. It is important here to keep track of the reading directions: contiguity with the same reading direction corresponds to preservation of the original genomic coordinates, while a change in reading direction indicates change of the order. Thus we distinguish preserved and inverted reading direction in our quantitative analysis.

Open reading frames (ORFs) are among the best-conserved features in the genome due to the strong selection pressures acting to preserve the corresponding proteins. As an immediate consequence we expect that ORFs are almost always preserved. This should be reflected also by the supergenome coordinates, i.e., blocks belonging to the same ORF should have only a small number (smaller then 5) of other blocks between them and retain their relative order. For higher eukaryotes, we cannot expect near perfect adjacency of coding blocks, however, because larger introns are subject to local rearrangements. To quantify the proximity of blocks of an ORF, the distances between all adjacent blocks are determined as described above and their absolute values are added up to yield a single characteristic value. In addition we count the number of exons that are “broken up” in the sense that consecutive pieces do not have consecutive coordinates or are placed in reverse order in the supergenome. Coding genes and exons are taken as annotated for the corresponding genomes. We note that in particular for large, intron-rich genomes such as the insect data set $\mathbf {F}$ this is an additional source of errors.

## Results and discussion

We have devised a heuristic algorithm to extract a common coordinate system for a supergenome from a genome-wide multiple sequence alignment. The procedure has been tested on three alignments of very different size and difficulty: an easy instance comprising four closely related bacterial species, an intermediate size problem composed of seven yeast genomes, and the alignment of 27 insect genomes as the most difficult instance.

### Performance of individual components

The heuristic algorithm outlined above is composed of several largely independent components. It is of interest, therefore to consider their relative impact on the final results. We find that most edges are removed by the mini-cycle remover, with a small contribution of Algorithm GR. On the other hand, the largest reduction of the vertex set is due to the merges identified by the closed DAG simplifier. More quantitative information is compiled in Figs. 6,  7 and 8 and in Additional file 1: Section 8. The simplifiers reduce the graph size by about an order of magnitude in both the number of vertices and edges, reducing it in size and complexity to a point where the seriation heuristic operates efficiently. The relative improvement is smallest for the bacterial data set.

Since the Colored Multigraph Betweenness Problem cannot be solved exactly in reasonable time for instances with sizes that are of interest for our application at hand, we cannot measure performance relative to the exact solution. The multigraphs obtained from real-life alignments contain a large number of conflicting edges. In the most difficult data set, $\mathbf {F}$, for instance, the final order keeps more than 95% of the initial edges.

### Quality of supergenome coordinate systems

The quality of the coordinate systems strongly depends on the quality of the input alignments. A detailed discussion of issues with the input alignments can be found in Additional file 1: Section 8. Here, we focus on an assessment of the coordinate systems themselves.

In order to check the overall quality of the solution we compute a betweenness graph from the supergenome coordinate systems. This is done by starting with a graph without edges. First, all edges that are supported by the total order of the supergenome are added. This is followed by edges that contradict the total order but do not create contradicting betweenness triples. Note that this graph is not necessary optimal but a good approximation that can easily be computed. The edge set of this graph is compared to the edge set of the initial graph. Good solutions are expected to retain most of the edges. For the three data sets we find that 95.3%, 97.5%, and 99.4% of the edges are retained in data sets $\mathbf {B}$, $\mathbf {Y}$, and $\mathbf {F}$, respectively.

**Fig. 9:**
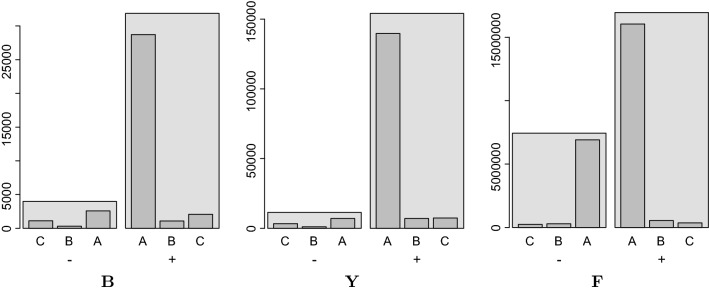
Distribution of block-wise distances of consecutive alignment blocks in the original genomes. Data are shown separated for inverted (−) and preserved (+) orientation of consecutive blocks (light gray). As expected, the number of inverted blocks increases with the difficulty of the input alignment. In particular, there is a substantial number of local inversions in the insect data set $\mathbf {F}$. Both the inverted (−) and preserved (+) bin are subdivided further into a bin of adjacent blocks (A), blocks with a distance of 1–5 blocks (B), and more distantly placed blocks (C), in the supergenome

The distribution of block-wise distances in the supergenome of alignment blocks that are consecutive in the original genome serves as a simple measure of preserved synteny. The results are summarized in Fig. 9 and presented in full detail in Additional file 1: Section 8. Another measure is how many of the input orders are preserved. To measure this we consider every alignment block and all successors from the different genomes. For the bacterial data set $\mathbf {B}$ $89\%$ of the successors preserve the order and $80\%$ also preserve the adjacency. For the yeast data set $\mathbf {Y}$ we observe that $93\%$ of the successors preserve the order and $84\%$ also preserve adjacency. This is a very encouraging result, taking in mind that every true genome rearrangement necessarily introduces at least one non-adjacency. Even in the much larger and more difficult insect set $\mathbf {F}$ we still find $70\%$ of the successors preserve the order and $66\%$ also preserve adjacency. The overwhelming majority of non-contiguous successors are placed in the adjacent but order-reversed position, reflecting the level of local rearrangements in the insect data set. This is entirely reasonable given the much larger number of species and their larger phylogenetic depth compared to the yeast data. Taken together, these numbers already indicate that the supergenome coordinates are meaningful and indeed are likely a useful starting point for large-scale multi-genome comparisons.

**Fig. 10:**
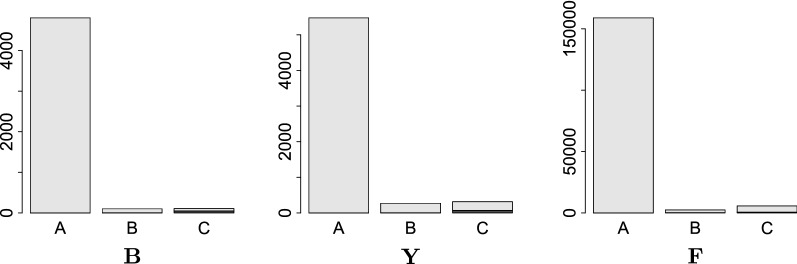
Distribution of block-wise distances between alignment blocks that contain ORFs for the bacteria and yeast data set. For the insect data invidual exons are considered since rearrangements as well as alignment errors within introns are not infrequent. The black bar indicates the number of broken ORFs/exons. The data is binned in three distance ranges: a distance of 0 (A), a distance of 1–100 (B), and a distance larger than 100 (C)

Restricting our attention to coding sequences yields a more stringent quality measure, as shown in Fig. 10. As bacteria have essentially no introns, we expect that nearly all blocks belonging to the same ORF retain both adjacency and order. In the bacterial data set $\mathbf {B}$ $96\%$ of the ORFs are in one stretch with no interruption and less then $1\%$ of the ORFs are broken. Since yeasts have few and short introns [74] we expect that data set $\mathbf {Y}$ is also very well-behaved in this respect. It contains 6062 ORFs annotated for *Saccharomyces cerevisiae*. Of these, 5474, i.e., $90\%$, are consistently represented in the coordinate system. An additional 272 ORFs, about $5\%$, have a distance of less then 100 blocks between them. Only 73, i.e., a bit more than $1\%$ of the ORFs are broken. For *Drosophila melanogaster* are 167, 051 exons annotated, and part of the alignment $\mathbf {F}$. Due to large and abundant introns the analysis is based on individual exons rather than complete ORFs for set $\mathbf {F}$. We observe that $95\%$ of the ORFs/exons are consistently represented. Only 779, about $0.5\%$, are broken. Overall, thus, the supergenome coordinates behave very well for all three data sets.

As a specific example we consider the genes of the yeast TCA cycle [75] in more detail. It is one of the best-studied enzyme systems and known to be essential in *S. cerevisiae*. There, it comprises 20 genes [76–79], all of which are contained at least partially in the initial set of alignment blocks in the yeast data set $\mathbf {Y}$. Only nine genes are included in their entirety, however. Seven of these nine are represented colinearly in single blocks. The alignments for KGD2 and SDH2 cover multiple MSA blocks and there is intervening genomic sequence in the input alignment, leading to non-contiguous placement in the final coordinate system. The alignment blocks referring to the remaining 11 genes are difficult to analyze and may contain misassigned sequences. This example, similar to several other loci, suggests that the quality of the input alignment rather than the complexity of the betweenness problem is the limiting factor for the construction of supergenome coordinate systems.

## Conclusion

In this contribution we have shown that the problem of computing a common coordinate system for supergenomes with the Colored Multigraph Betweenness Problem is NP-hard. It belongs to a class of relatively poorly studied betweenness problems for which few efficient heuristics have been developed so far. We introduced here several local simplification rules that can be applied iteratively to reduce the problem. It is important to note these reduction steps are only heuristics and do not guarantee optimal solutions. In conjunction with a simple serialization approach for the residual graph, they nevertheless yield practically useful results with acceptable computational efforts.

The most immediate application of the supergenome sorting problem is the direct comparison of genome annotations for multiple genomes. Hence it constitutes a prerequisite for comparative genome browsers. We have applied our approach to three real-life data sets of different sizes and difficulties. Our results indicate that practically useful coordinatizations can be computed. The computational requirement of the method scales favorably so that in principle even the largest genome-wide multiple sequence alignments could be processed.

The present study, however, also highlights the shortcomings of currently available genome-wide multiple sequence alignments [80, 81]. The issue is not only the relatively moderate coverage with alignment blocks that contain at least most of the species under consideration, but also the substantial fractions of alignment blocks that have been removed from our data set due to likely artefactual sequences. We have therefore not attempted to analyze the UCSC 100-way vertebrate alignments, since these data are even more complex than the insect data due to the very large number of paralogs introduced by genome duplications.

Synteny, i.e., the preservation of relative genome order, is in general a good predictor for homology. This fact suggests to use the common coordinate system to identify likely homologous regions that are not included in the initial alignment blocks. These could then be (re)aligned at sequence level and included in a revised multiple sequence alignment. This, in turn, could yield an improved common coordinate system. The systematic improvement of genome-wide alignments, albeit an interesting and extremely useful endeavor, is beyond the scope of this contribution.

Possible improvements of the approach taken here are conceivable in at least two directions. First, one may consider a hybrid algorithm that solves subgraphs with a dominant backbone use the method discussed in [29]. As discussed, we assume that large parts of the global graph structure are not amenable to such a solution, but it is also reasonable to assume that gene regions under strong conservation pressures can be solved fairly easily using a local backbone-based approach. A second venue of research is concerned with the determination of the final backbone order. Depending on the phylogenetic range under investigation, the ancestral gene order would provide a useful backbone based on the phylogeny of the species involved in the alignment.

## Additional file

**Additional file 1.** Additional Text covering some proofs, details on the data set used for evaluation, pseudocode of the algorithms described in the main text, and additional benchmark data.
