# Supplementary material for: Coordinate systems for supergenomes
Source: Algorithms Mol Biol. 2018 Sep 24;13:15. doi: 10.1186/s13015-018-0133-4 (PMC6151955; doi:10.1186/s13015-018-0133-4)
Supplement: Supplementary file 1 — Additional file 1. Additional Text covering some proofs, details on the data set used for evaluation, pseudocode of the algorithms described in the main text, and additional benchmark data. [file 13015_2018_133_MOESM1_ESM.pdf]

RESEARCH

Coordinate Systems for Supergenomes Supplemental Material

Fabian Gärtner[1,2*], Christian Höner zu Siederdissen[2,3], Lydia Müller[4,3,1] and Peter F Stadler[2,3,1,5,6,7,8,9]

*Correspondence: fabian@bioinf.uni-leipzig.de
[1]Competence Center for Scalable Data Services and Solutions Dresden/Leipzig, Universität Leipzig, Augustusplatz 12, D-04107 Leipzig, Germany
[2]Bioinformatics Group, Department of Computer Science, Universität Leipzig, Härtelstraße 16–18, D-04107 Leipzig, Germany
Full list of author information is available at the end of the article

Contents

1 Theory **2**
1.1 Betweenness Problem . . . 2
1.2 Colored Multigraph Betweenness Problem . . . 2

2 Datasets **4**
2.1 **B** Data Set: 4way Salmonella . . . 4
2.2 **Y** Data Set: 7way Yeast . . . 4
2.3 **F** Data Set: 27way Insect . . . 5

3 Filter **6**
3.1 Length Filter . . . 6
3.2 Score Filter . . . 6
3.3 Overlap Filter . . . 7

4 Simplifier **10**
4.1 Supperbubble Simplifier . . . 11
4.2 Sink and Source Simplifier . . . 11
4.3 Mini-Cycle Complex Remover . . . 11

5 Minimum Feedback Arc Set problem **17**

6 Topological Sorting **19**

7 Optimization **21**

8 Result **26**
8.1 Data distribution . . . 26
8.2 Graph edit statistic . . . 28
8.3 Graph properties . . . 29
8.4 Successor statistic . . . 30
8.5 ORF statistic . . . 32
8.6 Betweeness statistic . . . 33

1 Theory

1.1 Betweenness Problem

Betweenness Decision Problem: *Given a finite set X and a collection $\mathscr{C}$ of triples from X, is there a total order on X such that $\forall(i, j, k) \in \mathscr{C}$ either $i < j < k$ or $i > j > k$?*

Lemma 1 *The* Betweenness Decision Problem *is NP-complete.*

Proof The proof can be found in Opatrny et al. [1]. □

Betweenness Optimization Problem: *Given a finite set X and a collection $\mathscr{C}$ of triples from X, what is the maximum subset $C \subseteq$ from $\mathscr{C}$ such that there is a total order on X such that $\forall(i, j, k) \in C$ either $i < j < k$ or $i > j > k$?*

Algorithm 1: The algorithm that reduce the decision to the optimization problem, where "BOP(X, C)" applies the Betweenness Optimization Problem.

function reduce;
Input : X and $\mathscr{C}$
Collection m = BOP($X, \mathscr{C}$);
return $|m| == |X|$

Lemma 2 *The* Betweenness Optimization Problem *is NP-hard.*

Proof The Betweenness Decision Problem can reduce in constant time to the Betweenness Optimization Problem. An algorithm to do this is given in Algorithm 1. □

1.2 Colored Multigraph Betweenness Problem

Colored Multigraph Betweenness Decision Problem: Given the colored multigraph $\hat{\Gamma} = (V, E)$, is there a total order on V such that $\forall(i, j, k) \in \mathscr{C}(\hat{\Gamma})$ either $i < j < k$ or $i > j > k$?

Lemma 3 *The* Colored Multigraph Betweenness Decision Problem *is NP-complete.*

Proof The Colored Multigraph Betweenness Decision Problem is in NP. This is proofed by the polynomial time reduction to the Betweenness Decision Problem in Algorithm 2.
The problem is NP-hard.This is proofed by the polynomial time reduction of the Betweenness Decision Problem to the Colored Multigraph Betweenness Decision Problem. An algorithm to do this is given in Algorithm 3.
After the problem is NP-hard and in NP it is NP-complete. □

Algorithm 2: The algorithm that reduce the COLORED MULTIGRAPH BETWEENNESS DECISION PROBLEM to the BETWEENNESS DECISION PROBLEM, where $G(\Gamma)$ are all colors in Γ, E_g is the set of all edges with the color g and "BDP$(V, \mathscr{C})$" applies the BETWEENNESS DECISION PROBLEM.

```
function reduce;
Input    : Γ = (V, E)
Collection 𝒞 = Collection();
foreach g ∈ G(Γ) do
    foreach v ∈ V do
        foreach (i, v) ∈ E_g do
            foreach (v, j) ∈ E_g do
                𝒞.add((i, v, j))
            end
        end
    end
end
return BDP(V, 𝒞)
```

Algorithm 3: The algorithm that reduce the BETWEENNESS DECISION PROBLEM to the COLORED MULTIGRAPH BETWEENNESS DECISION PROBLEM, where "CMBDP(Γ)" applies the COLORED MULTIGRAPH BETWEENNESS DECISION PROBLEM.

```
function reduce;
Input    : X and 𝒞
Collection E = Collection() foreach g = (i, j, k) ∈ 𝒞 do
    Add edge (i, j) with color g to E;
    Add edge (i, j) with color g to E;
end
Γ = (X, E);
return CMBDP(Γ)
```

COLORED MULTIGRAPH BETWEENNESS PROBLEM: Given a colored multigraph $\hat{\Gamma} = (V, E)$, find a total order on V such that $E^* \subseteq E$ is maximal under the condition that $\forall (i, j, k) \in \mathscr{C}(V, E^*)$ either $i < j < k$ or $i > j > k$.

Lemma 4 *The* COLORED MULTIGRAPH BETWEENNESS PROBLEM *is NP-hard.*

Proof The COLORED MULTIGRAPH BETWEENNESS DECISION PROBLEM can reduce in constant time to the COLORED MULTIGRAPH BETWEENNESS OPTIMIZATION PROBLEM. An algorithm to do this is given in Algorithm 4. □

Algorithm 4: The algorithm that reduce the decision to the optimization problem, where "MBDP(Γ)" applies the COLORED MULTIGRAPH BETWEENNESS OPTIMIZATION PROBLEM.

```
function reduce;
Input    : Γ = (V, E)
Collection m = CMBOP(Γ);
return |E| == |m|
```

2 Datasets

One small data set of different Salmonella strains is created with Cactus [2]. Two larger data sets are downloaded from the UCSC genome browser website. They can be found here: http://hgdownload.soe.ucsc.edu/downloads.html

2.1 B Data Set: 4way Salmonella

This data set is created with Cactus [2]. The genomes of four Salmonella strains are aligned. They are described in Table 1. *Salmonella enterica Newport SL254* is used as reference genome for the alignment.

Table 1 An overview of the sequence data in the Salmonella alignment.

Name	Abbreviation	URL	Size	N50
Salmonella enterica serovar Agona SL483	SenAgo	SenAgo	4836638	4798660
Salmonella enterica serovar Dublin CT_02021853	SenDub	SenDub	4917459	4842908
Salmonella enterica serovar Heidelberg SL476	SenHei	SenHei	4983515	4888768
Salmonella enterica serovar Newport SL254	SenNew	SenNew	5007719	4827641

The Salmonella alignment has 13,416 blocks. Those blocks contain 50,932 sequences which have a combined total length of 18,047,456 nucleotides. This corresponds to 91% of the combined genome length of the genomes in the alignment.

The filters described in Section 3 are applied to the alignment. Statistical results are shown in Table 2. The result are significant different from the other data set due to the alignment method used for this data set. Cactus create no overlap and gives the blocks no score so that this two filters have no effect. On the other hand, cactus creates many small blocks. Thus, the length filter discards 30% of all blocks. However, the length of the discarded blocks sum up to only 0.4% of the nucletides in the alignment.

Table 2 An overview of the filters for the Salmonella alignment.

Filter	Blocks(b)	Sequence fragments(f)	Nucleotides(n)	b %	f %	n %
none	13416	50932	18047456	100	100	100
length	3986	15080	68141	30	30	0.4
score	0	0	0	0	0	0
overlap	0	0	0	0	0	0
all	3986	15080	68141	30	30	0.4

2.2 Y Data Set: 7way Yeast

The data set can be found here: http://hgdownload.soe.ucsc.edu/goldenPath/sacCer3/multiz7way. It consists of genome assemblies of seven yeast species. They are described in Table 3. *S. cerevisiae* is used as reference genome for the alignment.

Table 3 An overview of the sequence data in the yeast alignment.

Name	Abbreviation	URL	Size	N50
S. cerevisiae	sacCer3	sacCer3	12157105	813184
S. paradoxus	sacPar	sacPar	11872617	46034
S. mikatae	sacMik	sacMik	11470251	19428
S. kudriavzevii	sacKud	sacKud	11132834	11253
S. bayanus	sacBay	sacBay	11477549	24596
S. castelli	sacCas	sacCas	11242286	59644
S. kluyveri	sacKlu	sacKlu	10992590	8690

The yeast alignment has 49,795 blocks. Those blocks contain 275,484 sequences which have a combined total length of 71,517,259 nucleotides. This corresponds to 89.01% of the combined genome length of the genomes in the alignment.

The filters described in Section 3 are applied to the alignment. Statistical results are shown in Table 4.

Table 4 An overview of the filters for the yeast alignment.

Filter	Blocks(b)	Sequence fragments(f)	Nucleotides(n)	b %	f %	n %
none	49795	275484	71517259	100	100	100
length	6190	31423	134881	12	11	0
score	110	492	6311	0	0	0
overlap	13458	76141	18446796	27	28	26
all	19758	108056	18587988	40	39	26

2.3 F Data Set: 27way Insect

This data sets can be found here: http://hgdownload.soe.ucsc.edu/goldenPath/dm6/multiz27way/. It consists of 27 assemblies of insect species. They are described in Table 5.*D. melanogaster* is used as reference genome for the alignment.

Table 5 An overview of the sequence data in the Insect alignment. Some of the genomes are in 2bit format for details see here.

Name	Abbreviation	URL	Size	N50
D. melanogaster	dm6	dm6	143726002	25286936
D. simulans	droSim1	droSim1	142420719	22036055
D. sechellia	droSec1	droSec1	166577145	2123299
D. yakuba	droYak3	droYak3	165709965	21770863
D. erecta	droEre2	droEre2	152712140	18748788
D. biarmipes	droBia2	droBia2	169378599	3386121
D. suzukii	droSuz1	droSuz1	232923092	388966
D. ananassae	droAna3	droAna3	230993012	4599533
D. bipectinata	droBip2	droBip2	167263958	663995
D. eugracilis	droEug2	droEug2	156942009	976885
D. elegans	droEle2	droEle2	171267669	1714184
D. kikkawai	droKik2	droKik2	164292578	903682
D. takahashii	droTak2	droTak2	182106768	387676
D. rhopaloa	droRho2	droRho2	197375704	45514
D. ficusphila	droFic2	droFic2	152439475	1050541
D. pseudoobscura	droPse3	droPse3	152696384	12541198
D. persimilis	droPer1	droPer1	188374079	1869541
D. miranda	droMir2	droMir2	136728780	28826359
D. willistoni	droWil2	droWil2	235516348	4511350
D. virilis	droVir3	droVir3	206026697	10161210
D. mojavensis	droMoj3	droMoj3	193826310	24764193
D. albomicans	droAlb1	droAlb1	253560284	23589
D. grimshawi	droGri2	droGri2	200467819	8399593
Musca domestica	musDom2	musDom2	200467819	8399593
Anopheles gambiae	anoGam1	anoGam1	287805703	53272125
Apis mellifera	apiMel4	apiMel4	250287000	13219345
Tribolium castaneum	triCas2	triCas2	199682416	13894384

The insect alignment has 2,112,962 blocks which contain 36,139,620 sequences. They have a combined length of 2,172,959,429 nucleotides which correspond to 38.45% of the combined genome length of the genomes in the alignment.

The filters described in section 3 are applied to the alignments. The statistical result are shown in Table 6.

Table 6 An overview of the filters for the Insect alignment.

Filter	Blocks(b)	Sequence fragments(f)	Nucleotides(n)	b %	f %	n %
none	2112962	36139620	2172959429	100	100	100
length	531676	8362669	30024535	25	23	1
score	25557	384853	4074333	1	1	0
overlap	178882	3134544	285684545	8	9	13
all	736115	11882066	319783413	35	33	15

3 Filter

Noisy data and the alignment procedure make it necessary to curate the input data before calculating the common coordinate system. As part of the curation of input data sets some blocks are filtered out. Here, we give the pseudocode of the three filters already described in the main manuscript. Additionally, we show some results that explain the chosen parameters.

The filters are used to reduce the noise in the data. The noise has three main sources. Firstly, sequences may align by pure chance on each other. Secondly, multiz may produces small blocks that have no or almost no information in it. Thirdly, duplication events lead to sequences which are not different enough to align them unambigous and thus, lead to multiple alignments containing the same sequence for at least one species.

Filters do not discard blocks completely but mark the corresponding blocks and sequences as thrown out using the function `setThrowout` (). The graph generation algorithms and simplifiers (see below) check the thrown out flag using the function `isThrowout` (). Blocks and sequences for which the flag is set are skipped.

3.1 Length Filter

This filter targets the first and the second source for noisy data. Short blocks created by the aligner can be a result of random sequences. We filter out blocks with a length of at most 10 nucleotides. This size is used to discard random alignments but no useful information. Since the DNA consists of 4 nucleotides, there are 4^{10} possible sequences of length 10. This are 1.048.576 possibilities. Even the small yeast data set is 77 times longer than this number. Thus, it is clear that it is very likely that this sequences are likely the result of random alignments.

Each block has an attribute that shows the length of the corresponding alignment. Each sequence in this blocks at most as long as the alignment itself. Sequences might be shorter due to alignment gaps.

The pseudocode of the length filter is shown in Algorithm 5.

Algorithm 5: The algorithm that filters out too short blocks.

```
function filter (blocks);
Input  : blocks the list of Block that should be filtered
Output : The number of filtered Block
int sum = 0;
foreach Block b ∈ blocks do                          /* Check all Block */
    if b.isThrowout () then
        continue;
    end
    if b.length <= 10 then
        b.setThrowout ();
        sum++;
    end
end
return sum
```

3.2 Score Filter

Multiz may create alignments with bad scores as a side effect of the way how the blocks borders are determined. They likely contian no or not trustworthy information for the common coordinate system. The bad scored blocks are removed with

the score filter. In the current implementation, scores are normalized by alignment length and number of aligned sequences and a threshold of -30 is used as threshold for the filtering out a block.

The alignments scores that are given by the UCSC alignments are calculated on base of the BlastZ scoring scheme. So that the score filter is created with this scheme in mind. If another scheme is used, the constants has to adapted or the scores has to recalculated using the BlastZ scoring scheme.

Table 7 The HOXD70 scoring scheme after [3]. In the BlastZ score this used with affine gap scores where the opening penalty are 400 and the extension penalty is 30.

	A	C	G	T
A	91	−114	−31	−123
C	−114	100	−125	−31
G	−31	−125	100	−114
T	−123	−31	−114	91

The BlastZ scoring scheme is based on the HOXD70 scoring matrix [3] with affine gap scores. The scoring scheme is shown in Table 7. The gap opening penalty is 400 and gap extension penalty is 30. It is used to compute all pairwise alignment scores. For the scoring of the multiple alignments of the MAF-blocks, the sum-of-pair score is applied, i.e. all pairwise scores are computed and then summed up to form the MAF-Block score.

The pairwise alignment that contains no information is the alignment where both sequences are aligned completely to gaps. The score of such alignment with sequence length n and m would be:

$$\begin{aligned} & -400 - 30 \cdot (n-1) - 400 - 30(m-1) \\ = & -370 - 30 \cdot (n) - 370 - 30(m) \\ = & -740 - 30 \cdot (n+m) \end{aligned} \tag{1}$$

The alignment length then is $n + m$. This means that the normalized score of this is:

$$\begin{aligned} & \frac{-740 - 30 \cdot (n+m)}{n+m} \\ = & \frac{-740}{n+m} - 30 \end{aligned} \tag{2}$$

It is easy to see that for long sequences such an alignment would have a normalized score of around -30. These is the reason why every block with a score less then -30 most likely contain no useful information. Thus, blocks with a normalized score of at most -30 are discarded.

The score is saved as attribute of the block. The pseudocode of the score filter is shown in Algorithm 6.

3.3 Overlap Filter

One assumption of the golden genome is that the alignment is injective. However, multiz alignments are not injetive. Thus, this feature has to be actively created. this is done by filtering all overlapping sequences. Again, random alignments can be a

Algorithm 6: The algorithm that filter to short blocks.

```
function filter (blocks);
Input   : blocks the list of Block that should be filtered
Output  : The number of filtered Block
int sum = 0;
foreach Block b ∈ blocks do                          /* Check all Block */
    if b.isThrowout () then
        continue;
    end
    if b.score < −30 then
        b.setThrowout ();
        sum + +;
    end
end
return sum
```

problem. Especially, the exact start and stop position of an alignment on a genome is often not clear. Therefore, a small number of nucleotides may be used in two alignments. Discarding all overlaps would thus mean a big data loss. In particular, small overlaps are not a problem for the general idea and thus should be kept.

To allow this short overlapping ends, a overlap from up to 20 nucleotides is allowed on both ends. The explaination for this cutoff is again the combiantorical argument. There are 4^{20} possible sequences with 20 nucleotides. This number is larger then the size of the whole insect alignment. Thus, overlaps larger than 20 nucleotides indicate truely doublicated sequences.

Note that sequences in blocks shorter than 20 nucleotides may still overlap completely with other sequences without being filtered out. To avoid this, sequences completly overlapped by other sequences are filtered out. Hereby, sequences may be completely covered by the sequence from the previous block, the next block, or by the previous and next block.

Due to the fact that the sequences on one chromosomes are sorted by their start positions, the calculation of the overlap is a local problem. Overlaps with blocks before the current block and after the current block are calculated independently. The overlap filter marks the sequences as overlapping but not as thrown out to be able to distinguish them from sequences thrown out by previous filtering steps. In a later step, the marked sequences are thrown out. Algorithm 7 shows the pseudocode of the overlap filter.

Algorithm 7: The algorithm that mark overlapping sequences.

```
function filter (chr);
Input    : chr the Chromosome for which the Sequence shout be filtered
int end = 0;
for int i = 0; i < chr.length; i++ do
    Sequence seq = chr[i];
    if seq.isThrowout () then
        continue;
    end
    while end < i do
        if not chr[end].isThrowout () then
            if chr[end].stop > seq.start then
                break;
            end
        end
        end++;
    end
    int front = 0;
    for int j = end; j < i; j++ do
        int overlap = chr[j].stop − seq.start;
        front = max(front, overlap);
    end
    int back = 0;
    for int j = i; j < chr.length; j++ do
        int overlap = min(seq.stop − chr[j].start, chr[j].length);
        back = max(back, overlap);
        if overlap < 0 then
            break;
        end
    end
    if (front + back) >= seq.length then
        seq.setOverlap ();
    end
    if max(back, front) > 20 then
        seq.setOverlap ();
    end
end
```

4 Simplifier

For the graph simplification three simplifier are used. These are shown here as pseudocode.

The simplifier uses three non standard objects: the graph, the vertex, and the edge. The *graph* consists of vertices and edges and has the following functions:

- successor(vertex)
  Get the successors of the vertex.
- predecessor (vertex)
  Get the predecessor of the vertex.
- outDegree (vertex)
  Get the outDegree of the vertex.
- inDegree (vertex)
  Get the inDegree of the vertex.
- existsEdge (vertex;vertex)
  Check if there is an edge from the first to the second vertex.
- subgraph (list)
  Create the induce subgraph that contains all vertices in list.
- remove (vertex)
  Remove the vertex and all corresponding edges from the graph.
- remove (edge)
  Remove the edge from the graph.
- outgoingEdges (vertex)
  Get all edges that goes out of a vertex.
- incomingEdges (vertex)
  Get all edges that goes in a vertex.
- add (vertex,vertex, edge)
  Add the edge from the first to the second vertex to the graph.

Furthermore, one can also iterate over the vertices and edges of the graph.

A *vertex* can contain one or more alignment blocks. To mange this, the following functions are given:

- clear()
  Remove all blocks from the vertex
- addAll(list)
  Add all blocks in the list to the vertex.
- addFirst(vertex)
  Add all blocks in vertex to the front of the vertex.
- addLast(vertex)
  Add all blocks in vertex to the back of the vertex.

The *edge* stores among others a source and target vertex. Source and target vertex are read-only attributes of the edge:

- source
  Get the source of the edge.
- target
  Get the target of the edge.

Additionally, each edge has a color. This attribute links to the chromosome associated with the edge.

4.1 Supperbubble Simplifier

The pseudocode of the supperbubble simplifier is shown in Algorithm 8. It is strutuctured into three sub-tasks: searchDAG (Algorithm 9), sortDAG (Algorithm 11), and removeDAG (Algorithm 12). The searchDAG algorithm use the function checkPredecessor (Algorithm 10) which checks if all predecessors of a node are contained in a given DAG.

Algorithm 8: The algorithm that simplify the supperbubbles in the graph.

```
function simplify (g);
Input  : g theGraph that should be simplified
Output : The number of removed vertices
int sum = 0;
foreach Vertex v ∈ g do                              /* Check all Vertex  */
    Graph dag = searchDAG (v,g);                     /* Check Dag at v */
    if dag == null then                              /* No supperbubble */
        continue
    end
    boolean isCircle = sortDAG (v,dag,g);            /* Get local best order */
    if isCircle then                                 /* No DAG because of cycle */
        continue
    end
    sum += removeDAG (v,dag,g);                      /* Remove obsolete Vertex  */
end
return sum
```

4.2 Sink and Source Simplifier

The optimal position of sinks and source are behind and before the predecessor and successor, respectively. Algorithm 13 shows the complete implementation.

4.3 Mini-Cycle Complex Remover

The mini-cylce complex remover uses the following three objects:

- Complex
  The complex that contains a set of all connected mini-cycle. It can be iterated over the vertices in the complex and checked if a vertex is in the complex.
  - add(vertex)
    Add a new vertex to the complex
  - length
    The number of vertices in the complex
  - getBestAdjacency()
    Get the adjacency that has the best value
  - getBefore(vertex)
    Get all incoming adjacencies of the vertex.
  - getAfter(Vertex)
    Get all outgoing adjacencies of the Vertex.
  - remove(adjacency)
    Remove adjacency from the Complex.
- Order
  A partial order that contains all decisions that are made in a complex
  - addSuccessor(vertex,vertex)
    Add the order information that the first vertex is smaller then the second vertex.

Algorithm 9: The algorithm that find in a graph a supperbubble with the source v

```
function searchDAG (v, g);
Input    : v the Vertex that is the designated source of the supperbubble. g theGraph that
           should be simplified.
Output   : The found supperbubble
list next = g.successor (v) ;                     /* get the successor Vertex of v */
list dag;                                         /* init new list of Vertex */
dag.add (v);
boolean change = true ;                           /* true as long something changed */
while change do
  | change = false;
  | if next.length == 1 then                      /* Last vertex Found */
  |   | Vertex v2 = next[0];
  |   | if checkPredecessor (v2, g, dag) then     /* Valid sink */
  |   |   | dag.add (v2);
  |   |   | next.remove (v2);
  |   |   | break ;                               /* Dag complete */
  |   | else
  |   |   | return null ;                         /* Vertex has out of DAG connection */
  |   | end
  | end
  | foreach Vertex v2 ∈ next do                   /* Expand DAG */
  |   | if checkPredecessor (v2, g, dag) and g.outDegree (v2) == 0 then
  |   |   | dag.add (v2);                         /* Valid Expansion found */
  |   |   | next.remove (v2);
  |   |   | next.add (g.successor (v2));
  |   |   | change = true ;                       /* Dag has change */
  |   |   | break ;                               /* Restart loop */
  |   | end
  | end
end
if next.length ! = 0 then                         /* Check if DAG is closed */
  | return null
end
return g.subgraph (dag);
```

– valide(adjacency)
  Check if the direction of a adjacency is valid with the order information.
– isPredecessor(vertex,vertex)
  Check if the first vertex is a predecessor of the second vertex.

- Adjacency
  An adjacency in a complex. It bundles the information about all edges (both directions) of a mini-cycle and also contains direction information. It is possible to test if in the adjacency an edge with a specific chromosome exists.
  – source
    The source with respect to direction information
  – target
    The target with respect to direction information
  – getSwitch()
    Get the chromosomes of the edges that are contradicting the direction information.
  – switch(chromosome)
    Switches the direction of the edge that belongs to the chromosome.

The structure of the simplifier is shown in Algorithm 14. This function calls three sub-functions: findComplexe (Algorithm 15), decideComplex (Algorithm 16), and

Algorithm 10: The algorithm that checks if all predecessors are in the DAG

```
function checkPredecessor (v, g, dag);
Input  : v the Vertex for that is checked if all predecessors are in the DAG
Output : True if all predecessors in the DAG, false other wise.
foreach Vertex v2 ∈ g.predecessor (v) do                  /* All predecessors */
    if not v2 ∈ dag then
        return false;
    end
end
return true;
```

Algorithm 11: The algorithm that sort the DAG. If a cycle is found, null is returned and nothing is change in the graph. Otherwise add all vertices in v and add new edges from the sink in the DAG.

```
function sortDAG (v, dag, g);
Input  : v the Vertex that is the source of the DAF that is given as graph. Also the
         complete graph is given.
Output : True if a cycle is in the DAG, false other wise.
list sort = topoSort (dag);          /* Do topological sorting, see section 6 */
if sort == null then                                     /* A cycle is found */
    return true;
end
v.clear ();
v.addAll (sort);
foreach Vertex v2 ∈ dag do                              /* All Vertices in dag */
    foreach Edge e ∈ g.outgoingEdges (v2) do            /* All outgoing Edges */
        g.add (v,e.target,e);                                /* Add edge to v */
    end
end
return false;
```

removeEdges (Algorithm 18). The function switchDirection (Algorithm 17) is used by decideComplex to locally switch the direction of edges for a give chromosome.

Algorithm 12: The algorithm that remove all vertices other then v of the DAG out of the complete graph.

```
function removeDAG (v, dag, g);
Input  : v the Vertex that is the source of the dag that is given as Graph. Also the complete
         Graph is given.
foreach Vertex v2 ∈ dag do                              /* All Vertices in dag */
    if v2 != v then
        g.remove (v2)
    end
end
return false;
```

Algorithm 13: The algorithm that simplifies a source or a sink in the graph

```
function simplify (g);
Input   : g theGraph that should be simplified
Output  : The number of removed vertices
int sum = 0;
foreach Vertex v ∈ g do                                  /* Check all Vertex  */
    if g.inDegree (v) == 0 then                          /* Is a source */
        list suc = g.successor ();
        if suc.length == 0 then                          /* have only one successor */
            suc[0].addFirst (v);                         /* Add source before successor */
            g.remove (v);                                /* Remove source */
            sum+ = 1;
        end
    end
    if g.outDegree () == 0 then                          /* Is a sink */
        list pre = g.predecessor ();
        if pre.length == 0 then                          /* have only one predecessor */
            pre[0].addLast (v);                          /* Add sink after predecessor */
            g.remove (v);                                /* Remove sink */
            sum+ = 1;
        end
    end
end
return sum
```

Algorithm 14: The algorithm that remove all Mini-Cycle in the graph.

```
function simplify (g);
Input   : g the Graph that from which the mini cycle are removed
Output  : The number of removed edges
int sum = 0;
list complexe = findComplexe (g);                        /* Find all complexe */
foreach Complex c ∈ complexe do                          /* Check all Complex  */
    Order ord = decideComplex (c,g);                     /* Create order of vertices */
    sum+ = removeEdges (ord,c,g);                        /* Remove edges out of g */
end
return sum
```

Algorithm 15: The algorithm that find all Mini-cycle Complexes.

```
function findComplex (g);
Input  : g the Graph for which the Complex are found.
Output : The found Complex as a list
list complexes ;                                   /* init new list of Complex */
foreach Vertex v ∈ g do                            /* Check all Vertex */
    if v ∈ complexes then                          /* Already used Vertex */
        continue;
    end
    boolean change = true;
    Complex c ;                                    /* init new Complex */
    c.add (v);
    while change do
        change = false;
        foreach Vertex v2 ∈ c do                   /* Check all Vertices in Complex */
            foreach Vertex v3 ∈ g.successor (v2) do     /* Check successor */
                if g.existsEdge (v3, v2) then           /* Mini-Cycle */
                    if not v3 ∈ c then                  /* New Vertex */
                        c.add (v3);
                        change = true;
                    end
                end
            end
        end
    end
    if c.length > 1 then                           /* Complex found */
        complexes.add (c);
    end
end
return complexes
```

Algorithm 16: The algorithm that create a order of the complex and decides so which edge is kept.

```
function decideComplex (c, g);
Input  : g the Graph for which the Complex should decided.
Output : The found Order.
Order order ;                                      /* init new Order */
Adjacency start = c.getBestAdjacency ();           /* get best supported */
order.addSuccessor (start.source, start.target );
list next;                                         /* init new list of Adjacency */
switchDirection (start, c);                        /* Switch direction */
next.addAll (c.getBefor (start.source));
next.addAll (c.getAfter (start.target));
c.remove (start);
while next.length! = 0 do
    next.sort ();                                  /* sort after Adjacency.value */
    Adjacency nAd = next.pop ;                     /* get and remove best Adjacency */
    if order.valide (nAd) then                     /* Not violate order */
        order.addSuccessor (nAd.source, nAd.target );
        switchDirection (nAd, c);                  /* Switch direction */
        next.addAll (c.getBefor (nAd.source));
        next.addAll (c.getAfter (nAd.target));
    end
    c.remove (nAd);
end
return order
```

Algorithm 17: The algorithm that switch local the direction of a chromosome if a inversion is found.

```
function switchDirection (a, c);
Input    : a the Adjacency from which the direction should change in the Complex c.
list chroms = a.getSwitch ();                        /* get the inverse Chromosome  */
foreach Chromosome chr ∈ chroms do                   /* Check all Chromosome  */
    list bef;                                        /* init new list of Adjacency  */
    bef.addAll (c.getBefor (a.source));
    while bef.length! = 0 do
        Adjacency nAd = bef.pop ;                    /* get & remove first Adjacency  */
        if chr ∈ nAd then
            nAd.switch (chr);                        /* switch the Chromosome  */
            bef.addAll (c.getBefor (nAd.source));
        end
    end
    list after;                                      /* init new list of Adjacency  */
    after.addAll (c.getAfter (a.target));
    while after.length! = 0 do
        Adjacency nAd = after.pop ;                  /* get & remove first Adjacency  */
        if chr ∈ nAd then
            nAd.switch (chr);                        /* switch the Chromosome  */
            after.addAll (c.getAfter (nAd.target));
        end
    end
end
```

Algorithm 18: The algorithm that removes edges out of the graph. This destroy the Mini-Cycle.

```
function removeEdges (ord, c, g);
Input    : ord the Order that determine which edges in c are removed from g.
Output   : The number of removed Edges
int sum = 0;
foreach Vertex v ∈ c do                              /* Check all Vertex  */
    foreach Edge e ∈ g.outgoingEdges (v) do          /* Check Successor */
        if ord.isPredecessor (e.target,v) then
            g.remove (e);
            sum+ = 1;
        end
    end
end
return sum;
```

5 Minimum Feedback Arc Set problem

The order creation assumes a cycle-free graph. This is done by removing a minimum set of arcs that cause cycles. This Maximum Acyclic Subgraph or Minimum Feedback Arc Set problem is well-known to be NP-hard [4]. It is fixed parameter tractable (FPT) [5] but APX hard [6]. Nevertheless, fast, practicable heuristics have been devised, see e.g. [7, 8].

Given the size of our input graphs, we have to resort to linear-time heuristics. We use a modified version of `Algorithm GR` [7] because it is known to work particularly well on sparse graphs.

The idea is to create an order π out of the graph then remove all edges that goes from an vertex i to a vertex j if $\pi(j) < \pi(i)$ where $\pi(j)$ is the position of j in the order π. These guaranties an cycle free output graph. The tricky part is to create this order in a way that removes as less edges as possible.

For this, the order is fragmented in two parts, a front π_1 and a back part π_2, where then $\pi = \pi_1\pi_2$. To do so, a vertex removed from the graph and is either append to π_1 or added to the front of π_2. An simple observation is that all sources can be append to π_1 and all sinks can be added to π_2 without losing any edge.

The part where edges are lost is when no sources or sinks are left in the graph. Then a vertex v is removed with a maximal value for $d_{out}(v) - d_{in}(v)$ where d_{out} is the out degree of v and d_{in} is the in degree of v. With other words, it has many outgoing edges and less incoming edges. When v is append to π_1 only few edges are removed (incoming edges) and many edges are saved (outgoing edges).

We made only one modification to the algorithm. In the original `Algorithm GR`, a random vertex with the highest value is used. We try to use a vertex with a predecessor that is already append to π_1. This minimized the number of sources that are created without influencing the performance of the algorithm. In fact, it optimized the results of the heuristic on our data sets (Table 8).

Table 8 An overview of the heuristics that solve the FAS problem. The columns are the different data sets. The rows are the different algorithm. The first row provides the information of the original graphs of the data sets. The absolut number of edges are shown. The percentage of edges that are kept from the original graph are shown in brackets.

	Bacteria	Yeast	Insect
none	12285	160886	24248434
`Algorithm GR`	10860(88%)	103076(64%)	14847253(61%)
`Eades Serialization`	10871(88%)	105296(65%)	14913743(62%)

This modification is implemented in the function `Eades Serialization` that is shown in Algorithm 19. It has a time complexity of $O(|E| + |V|)$. The organization of the vertex degrees in an effective way is done by a class "VertexCategorizer". This class has all vertices in sortet in lists by there value and special functions that handle the removal of a vertex in an effective way. This removal handling is described also in the original paper [7]. The only interesting and changed function is the getMax() function that returns the next vertex that is removed when no sink or source in the graph exists.

The getMax() function is shown in Algorithm 20 and uses the function getMaxBucket() which returns the list of the vertices with the highest value. If this not exists, it returns null. Also the set "nosource" is used. It is a class member of "VertexCategorizer" and contains all vertices that can be removed without creating a source in the graph, i.e. a predecessor of such a vertex is already in π_1.

Algorithm 19: The algorithm that create a order. This order is used to removes edges out of the graph. This destroy all cycles in the graph.

```
function serialize (g);
Input  : g the Graph for which the cycle should be removed.
Output : The order with can be used to remove the cycles.
VertexCategorizer c = VertexCategorizer (g);     /* init the VertexCategorizer with g */
list s1;                                          /* init a new list of vertices */
list s2;                                          /* init a new list of vertices */
while true do
    if c.hasSink () then                          /* Remove sink first */
        Vertex v = c.getSink ();
        s2.addFirst (v);
        c.remove (v);
    end
    else if c.hasSource () then                   /* Remove source second */
        Vertex v = c.getSource ();
        s1.add (v);
        c.remove (v);
    end
    else                                          /* When no source or sink left */
        Vertex v = c.getMax ();
        if v =null then                           /* All vertices removed */
        end
        s1.add (v);
        c.remove (v);
    end
end
return s1 + s2;
```

Algorithm 20: The function from VertexCategorizer that decide which vertex is removed next from the graph.

```
function getMax ();
Output : The next vertex to remove from the graph.
list bucked =getMaxBucket ();
if bucked ==null then
    return null;
end
foreach Vertex v ∈ bucked do                      /* Check all Vertex */
    if nosource.contains (v) then
        return v;
    end
end
return bucked[0];
```

6 Topological Sorting

Topological sorting is performed using an adoption of a method presented by A. B. Kahn[9]. There are two variants. One that is the direct implementation of the method. It holds newly found sources in a queue that operates according to the first in, first out (FIFO) schema (Algorithm 21). The second variant uses a stack for the newly found sources, i.e. a queue operating according to the last in, first out (LIFO) schema (Algorithm 22). Both lead to valid topological orders with the difference in parts of the order where the order information is ambiguous. The second one creates the order that is better for our purpose. We generated results with both varaints to show the differences.

Both, queue and stack, have two functions and one attribute:

- add(Vertex)
  Add the vertex to the queue/stack
- pop()
  Get and remove the next outgoing element after the used scheme.
- lenght
  Get the number of elements in the queue/stack.

Algorithm 21: The simple Kahn algorithm that created Topologic sorting out of a graph.

```
function toposort (g);
Input   : g the Graph that should be ordered.
Output  : The order as list.
list order;                                              /* init a new list  */
Queue sources;                                          /* init a new Queue  */
foreach Vertex v ∈ g do                                 /* Check all Vertex  */
    if g.inDegree (v)==0 then                              /* Source found */
        sources.add (v);
    end
end
while sources.length! = 0 do
    Vertex v = sources.pop ();                            /* get next Source */
    order.add (v);
    list sucs = g.successor (v);                          /* get successors */
    g.remove (v);                                 /* Remove source out of graph */
    foreach Vertex v2 ∈ sucs do                          /* Check all Vertex  */
        if g.inDegree (v2)==0 then                          /* Source found */
            sources.add (v2);
        end
    end
end
return sum;
```

Algorithm 22: The advanced Kahn algorithm that created topological sorting out of a graph.

```
function toposort (g);
Input  : g the Graph that should be ordered.
Output : The order as list.
list order;                                                     /* init a new list  */
Stack sources;                                                  /* init a new Queue */
foreach Vertex v ∈ g do                                         /* Check all Vertex */
    if g.inDegree (v)==0 then                                   /* Source found */
        sources.add (v);
    end
end
while sources.length! = 0 do
    Vertex v = sources.pop ();                                  /* get next Source */
    order.add (v);
    list sucs = g.successor (v);                                /* get successors */
    sucs.sort ();              /* sort so that the highest inDegree comes first */
    g.remove (v);                                     /* Remove source out of graph */
    foreach Vertex v2 ∈ sucs do                                 /* Check all Vertex */
        if g.inDegree (v2)==0 then                              /* Source found */
            sources.add (v2);
        end
    end
end
return sum;
```

7 Optimization

The optimization has two main components: the validation of the order and the re-ordering.

The validation is done by the class RobinsonQuality. This can be done in a simple way of checking the neighborhood of every vertex and count the number of violations of the robinson inequation (see main manuscript). The neighborhood is defined by the number of hops (h). This means that a vertex is seen as neighbor if a path from one to the other exists that has at most length h. Consequently, the maximum neighborhood size is k^h with k being the maximal outdegree. So that this validation has a linear time complexity with respect to the number of vertices. For simplicity, this class is not shown in more detail.

Besides the validation of an exiting order, the evaluation of potential changes is required for an efficient optimization. Therefore, the class RobinsonQuality provides the function predictValue that takes the order and a change object as input and returns the number of violations of the robinson inequation taking the changes in the change object int account. This makes it possible to evaluate a movement before it is applied to the order.

The re-ordering is done in loop until no changes that optimize the order are found. This procedure is shown in Algorithm 23. It calls three other functions. First, a list of candidates are created by the function "initCandidates". These candidates are created only once. A candidate consists of two vertices. It is iterated through the candidates and two changes are tried for each of them. The changes are found with the functions "moveToFront" and "swapSiblings". The best non-overlapping changes is finally applied to the order.

The candidates are created by finding pairs of siblings. Two vertices are siblings if they have a direct predecessor in common or they are both sources. The function that creates that candidates is shown in Algorithm 24.

Finally, we will describe the two move functions: moveToFront and switchSiblings. This two function uses three help functions. The first one is called "checkPreIsNot-Between". It checks if a predecessor is in the same block and if a predecessor is between the blocks. This is shown in Algorithm 25. The second function is "check-PreInBlock" and checks if a predecessor is in the same block (Algorithm 26). The last function is "checkSucIsNotBetween". It checks if a successor is between the blocks and is shown in Algorithm 27.

The function "moveToFront" performs a move to front change on the order. This means that a block of vertices after the source position is moved to the target position. The moved block is as large as possible i.e. all vertices after the source position is added to the block as long no predecessor is between the target position and the source position. The function is shown in Algorithm 28.

The second function, "swapSiblings", performs a swap on the order. This means that a block of vertices after the source position is swapped with a block of vertices after the target position. The blocks are as large as possible but connected. Connected means that, beside the first vertex, every other vertex has a predecessor in the block. The function is shown in Algorithm 29.

Algorithm 23: The basic sort algorithm. That sort the order until no optimization is found.

```
function sort (order, quality);
Input  : order the order that is optimized, quality RobinsonQuality to check the quality of
         the order
Output : The order as list.
list candidates = initCandidates (order);                        /* get move condidates */
list changes;          /* init a new list for all changes that optimize the order */
repeat
    changes.clear ();
    foreach Vertex (v, w) ∈ candidates do                         /* Check all candidates */
        int target = order.getPosition (v);                        /* get target position */
        int source = order.getPosition (w);                        /* get source position */
        if target > source then  /* Make source target position<source position */
            (target, source) = (source, target);                 /* Swap target and source */
        end
        Change c = moveToFront (order, quality, target,source);
        if c!=null && c.val < quality.val then                      /* Optimization found */
            changes.add (c);
        end
        c = swapSiblings (order, quality, target,source);
        if c!=null && c.val < quality.val then                      /* Optimization found */
            changes.add (c);
        end
    end
    changes.sort ();                    /* sort after val i.e. greatest Optimization */
    list appChanges;                              /* init a new list to prevent overlap */
    foreach Change c ∈ changes do                                          /* outerloop */
        foreach Change applied ∈ appChanges do
            if applied.overlap (c) then               /* overlap with applied Change  */
                continue outerloop;                     /* continue with next Change */
            end
        end
        if quality.predictValue (order,c)< quality.val then
            order.apply (c);                                            /* apply Change */
            appChanges.add (c);
            quality.validate (order);                                     /* update val */
        end
    end
until changes.length == 0;
return order
```

Algorithm 24: The initialization of all valid moves in the order.

```
function initCandidates (order);
Input   : order the order that is optimized.
Output  : A list of the tuples that represent the move candidates
list sources;                                          /* init a new list  */
foreach Vertex v ∈ order do                            /* Check all Vertex  */
    if order.getPre (v).length==0 then                  /* Source found */
        sources.add (v);
    end
end
list candidates;                                       /* init a new list  */
for i = 0;i < sources.length;i++ do                    /* Add sources */
    for j = i + 1;j < sources.length;j++ do
        candidates.add ((sources[i], sources[j]));
    end
end
foreach Vertex v ∈ order do                            /* Add siblings */
    suc = order.getSuc (v);
    for i = 0;i < suc.length;i++ do
        for j = i + 1;j < suc.length;j++ do
            candidates.add ((suc[i], suc[j]));
        end
    end
end
return candidates
```

Algorithm 25: The test if a predecessor is between the target and source. Also a test if a predecessor is in the source block.

```
function checkPreIsNotBetween (order, targetP, vertexP, sourceP);
Input   : order the order that is optimized, targetP the position where the source block is
          moved. vertexP the position of the vertex for theme the predecessors are checked.
          sourceP the position where the source block started.
Output  : 0 when a predecessor is between targetP and sourceP. 1 if no predecessor
          between targetP and vertexP exists. 2 if only a predecessor between sourceP and
          vertexP exists.
if vertexP < order.length then
    return 0;
end
int ret = 1;
for int predeccesorP ∈ order.getPrePositions (vertexP) do
    if predeccesorP ≥ sourceP then
        ret = 2;
    end
    else if predeccesorP ≥ targetP then
        return 0;
    end
end
return ret
```

Algorithm 26: The test if a predecessor is in a block.

```
function checkPreInBlock (order, startP, vertexP);
Input   : order the order that is optimized, startP the position where the block is started.
          vertexP the position of the vertex for them the predecessors are checked.
Output  : true when a predecessor is between startP and vertexP.
for int predeccesorP ∈ order.getPrePositions (vertexP) do
    if predeccesorP ≥ startP then
        return true;
    end
end
return false;
```

Algorithm 27: The test if a predecessor is in a block.

```
function checkSucIsNotBetween (order, vertexP, targetP, endP);
Input  : order the order that is optimized. vertexP the position of the vertex for them the
         successor are checked. targetP the position where the block should move to. endP
         the position where the block ends.
Output : false when a successor is between endP and targetP.
for int successorP ∈ order.getSucPositions (vertexP) do
    if endP < predeccesorP ≤ targetP then
        return false;
    end
end
return true;
```

Algorithm 28: The algorithm that calculate for two siblings a move to the front Change

```
function moveToFront (order, quality, target, source);
Input  : order the order that is optimized, quality RobinsonQuality to check the quality of
         the change. The target as position and the source as position.
Output : The Change object that represent the possible move to front change.
int endOfBlock = source − 1;
while true do
    if endOfBlock + 1 == order.length then
        break;
    end
    endOfBlock++;
    if checkPreIsNotBetween (target, endOfBlock, source)==0 then
        endOfBlock–;
        break;
    end
end
if endOfBlock < source then
    return ;
end
Change c =Change (target, source, endOfBlock);
c.val = quality.predictValue (c);
return c
```

Algorithm 29: The algorithm that calculate for two siblings a swap Change

```
function swapSiblings (order, quality, target, source);
Input  : order the order that is optimized, quality RobinsonQuality to check the quality of
         the change. The target as position and the source as position.
Output : The Change object that represent the possible swap change.
int endOfSourceBlock = source;
if checkPreIsNotBetween (target, endOfSourceBlock, source)==0 then
    return ;
end
while true do
    if endOfSourceBlock + 1 == order.length then
        break;
    end
    endOfSourceBlock++;
    if checkPreIsNotBetween (target, endOfSourceBlock, source)!=2 then
        endOfSourceBlock–;
        break;
    end
end
int endOfTargetBlock = −1;
for int i = target + 1; i < source; i++ do
    if not checkPreInBlock (target, i) then
        endOfTargetBlock = i − 1;
        break;
    end
end
if  endOfTargetBlock == −1 then
    return null;
end
for int i = target; i ≤ endOfTargetBlock; i++ do
    if not checkSucIsNotBetween (i, source, endOfTargetBlock) then
        return null;
    end
end
Change c =Change (target, endOfTargetBlock, source, endOfBlock);
c.val = quality.predictValue (c);
return c
```

8 Result

8.1 Data distribution

As a simple statistical quality assessment, we calculated the coverage of the reference species of the alignments with alignment blocks. To quantify positional effects, the coverage is assessed for sliding windows along the genome. We furthermore weight the coverage with number of different species in the repsective blocks. These can be done before and after the filtering. Plotting the resulting data into the same coodinae system enables a direct comparison of the coverage by the genome graph before and after filtering. it allows to conclude whether filtering affects specific genome positions more than others.

The plots are generated for data set **Y** by using *Saccharomyces cerevisiae* as reference and for **F** by using *Drosophila melanogaster* as reference. The results show that for data set **Y** the coverage is in general high. The filtering leads to local loss of coverage but change nothing on the general high coverage. In the case of data set **F**, it is different. Here, six genomic regions aleady have a low coverage before filtering (end of 2L,beginning of 2R, end of 3L, begining of 3R, end of X and complete Y). Those genomic regions overlap with repeat regions of the chromosomes. Since repeat regions are very similar to each other and therfore hard to align, it is not surprising to observe a low coverage for those regions when using an alignment technique as done for the data set. For the remaining genome, the result is the same as by the data set **Y**. It has a high coverage for the graph before filtering and the filtering only lose local coverage.

After the filtering, the input alignments are transfromed into the supergenome graph and further cacluations only make use of the supergenome. This graph is then modified in three steps: first run of simplifer, apply FAS algorithm, and second run of simplifer. Four simplfier are applied to the graph in the fist run: the mini-cycle remover, the source/sink simplifier, and supperbubble simplifier. In the second run, the mini-cycle remover is replaced by the advance source/sink simplifier.

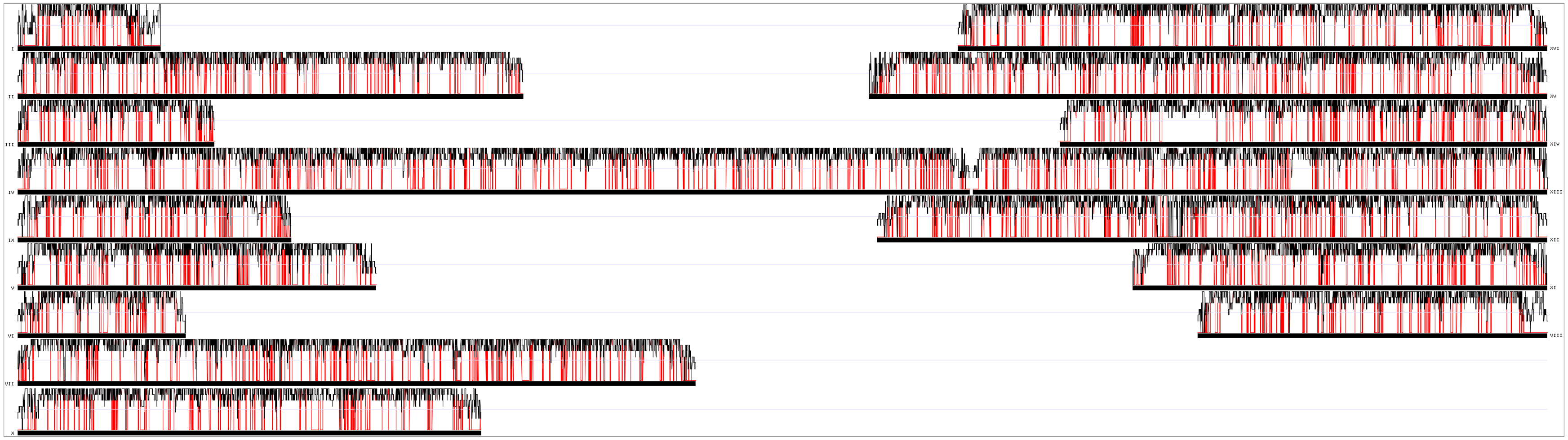

(a)

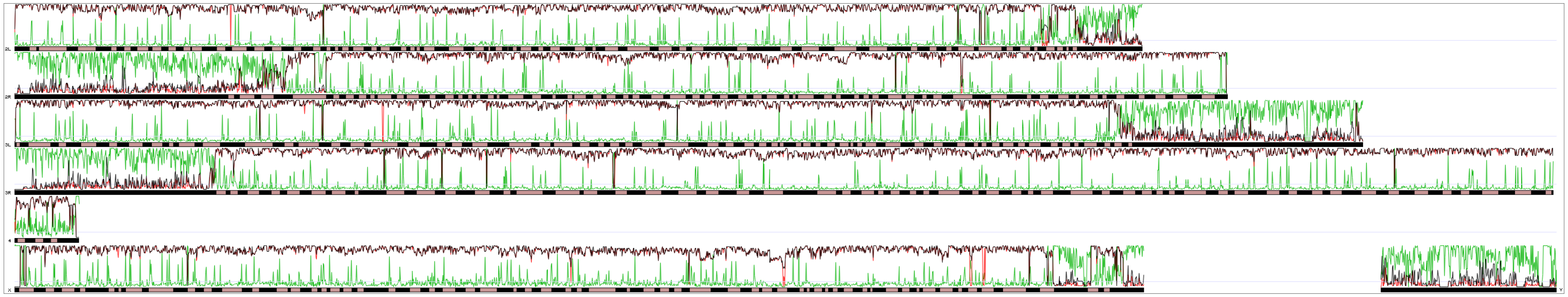

(b)

Figure 1 The data distribution on the reference genomes for data set $\mathbf{Y}$ (a) and data set $\mathbf{F}$ (b). The black line is the data distribution before any filter is used and the red line after all filters are applied to the data set. The y axis shows how many different species are contained in the alignment blocks at this position. So it is in (a) a value between 0 and 7 and in (b) a value between 0 and 27. To make the information more readable the information is given for regions not for every nucleotide. In case of data set $\mathbf{Y}$, 100nt are one data point and in case of data set $\mathbf{F}$ are 10000nt one data point. In (b) also in green the repeats after RepeatMasker are shown.

8.2 Graph edit statistic

For every graph, three edit steps are performed. The first run of simplifier, the remove of all cylces (FAS), and the second run of simplifier. All these steps remove edges from the graph. The Mini-Cycle Remover and FAS remove edges to break cycles in the graph. In the remaining cases, edges are lost as a side effect of the merging of connected vertices and the translation of the edges that connect them. The different steps are repeated by the edit tools several times. To get an idea of the time consumption of each tool in every step, a example time consumption is given. This is the result of an example run on a machine with a Intel(R) Xeon(R) CPU with 2.27GHz and enough RAM to fit the problem size in it. However, the exact numbers are not important. It rather provides an impression how long each step takes compared with the other steps. Moreover, it shows that the simplifier can be calculated even for big data sets in a feasible time on a normal machine.

The results are shown in Table 9, 10, and 11. Here, the three steps are indicated by horizontal lines. For every tool, we list how many vertices and edges have been removed along with the used time and the number of applications (number distinct position at which the tool could be used). For data set **F**, it is remarkable that most of the compute time is used in one big mini cycle complex. The complex has 896,082 vertices and it take 10,144 seconds to decompose it.

Table 9 The overview of the graph editors in data set B.

Edit tool	Removed Vertices	Removed Edges	Time in seconds	number of applications
Mini-Cycle Remover	0	2995	0.17	3
Source/Sink Simplifier	4	13	0.03	21
supperbubble Simplifier	5844	20550	0.50	21
FAS	0	1414	0.05	1
Advance Source/Sink Simplifier	67	197	0.05	14
Source/Sink Simplifier	3	6	0.01	14
supperbubble Simplifier	546	1436	1.27	14

Table 10 The overview of the graph modifications in data set Y

Edit tool	Removed Vertices	Removed Edges	Time in seconds	number of applications
Mini-Cycle Remover	0	62591	1.3	5
Source/Sink Simplifier	30	51	0.088	25
supperbubble Simplifier	23087	74977	1.3	25
FAS	0	1888	0.07	1
Advance Source/Sink Simplifier	125	465	0.17	9
Source/Sink Simplifier	143	346	0.014	9
supperbubble Simplifier	2257	7396	0.63	9

Table 11 The overview of the graph modifications in data set F.

Edit tool	Removed Vertices	Removed Edges	Time in seconds	number of applications
Mini-Cycle Remover	0	10620262	10439.68	10
Source/Sink Simplifier	41	99	12.54	48
supperbubble Simplifier	1033047	10282577	397.38	48
FAS	0	55286	6	1
Advance Source/Sink Simplifier	3505	25258	17.42	18
Source/Sink Simplifier	1641	7243	2.35	18
supperbubble Simplifier	85002	842444	96.91	18

8.3 Graph properties

The graph editing steps create different temporary graphs. After each step, some core information of the temporary graphs are collected. This are the number of vertices, edges, and connected components (C. C.), the median in-degree (In-Degree) and median out-degree (Out-Degree), the mean number of blocks in vertex (Mean B.), and the maximal number of blocks. As a reference, we calculated this values also for the original graph. The three temproary graphs are: the graph after the first run of simplifier (Simplfier 1), the graph after the FAS-algorithm is applied (FAS), and the graph after the second run of simplifier (Simplfier 2).

The data is shown in Table 12,13, and 14. In case of data set **B**, the second connected component is the third plasmid of *Salmonella enterica Newport SL254* that is with less then 4k nucleotide very small and is aligned with nothing. In case of data set **F**, the second connected component is created by the FAS algorithm. That can happened since the FAS algorithm is a heuristic. However, it contains only a handful vertices and is simplified to one vertex in the next step.

Table 12 The overview of the graph in data set B.

Graph	Vertexes	Edges	C. C.	In-Degree	Out-Degree	Mean B.	Max B.
Orignal	9430	35843	2	4	4	1.00	1
Simplfier 1	3582	12285	2	4	4	2.63	133
FAS	3582	10871	2	3	3	2.63	133
Simplfier 2	2966	9232	2	3	3	3.18	133

Table 13 The overview of the graph in data set Y.

Graph	Vertexes	Edges	C. C.	In-Degree	Out-Degree	Mean B.	Max B.
Orignal	30580	164114	1	6	6	1.00	1
Simplfier 1	7463	26495	1	4	4	4.10	102
FAS	7463	24607	1	3	3	4.10	102
Simplfier 2	4938	16400	1	3	3	6.19	122

Table 14 The overview of the graph in data set F.

Graph	Vertexes	Edges	C. C.	In-Degree	Out-Degree	Mean B.	Max B.
Orignal	1383449	24382316	1	18	18	1.00	1
Simplfier 1	350361	3479378	1	10	10	3.95	878
FAS	350361	3424092	2	10	10	3.95	878
Simplfier 2	260213	2549147	2	10	10	5.32	1054

8.4 Successor statistic

Most sequences have one successor sequence in the data set. These successor sequence is the sequence that follows the sequence on the contig. Note that successor depends on the reading direction of the sequence. If it is negative, then the successor sequence is in the contig reading direction direct before the sequence. If the reading direction is positive, the successor is the sequence that is in the contig reading direction direct behind the sequence. These sequences belong to blocks that are contained in the resulting order. So, the distance in blocks between the sequencen itself and it's successor can be calculated using the order. The distance is calculated in form that the number of blocks between them counted. So if they are adjacent they have a distance of zero. The data is disassembled in positive distances where the successor block is behind and in negative distances where the successor is before the sequence. Since the contig reading direction is arbitrary, we use the reading direction creating more positive distances for a contig.

The data is shown in Table 15,16, and 17. The data is binned in different distance ranges. Different ranges and all successors are evaluted for each direction. The absolute numbers as well as the fraction of all successors in the data set is given.

Table 15 The succesor distances in data set B.

Direction	Distance	Absolute	Fraction
+	all	31863	0.8889601874843066
+	0	28713	0.8010769187846999
+	1 − 5	1074	0.029964009709008733
+	6 − 20	331	0.009234718076053902
+	21 − 100	341	0.009513712579862176
+	101 − 1000	375	0.010462293892810311
+	> 1000	1029	0.028708534441871495
−	all	3980	0.11103981251569343
−	0	2570	0.07170158747872667
−	1 − 5	306	0.008537231816533214
−	6 − 20	68	0.00189716262589627
−	21 − 100	122	0.0034037329464609548
−	101 − 1000	187	0.005217197221214742
−	> 1000	727	0.02028290042686159

Table 16 The succesor distances in data set Y.

Direction	Distance	Absolute	Fraction
+	all	154113	0.9310276082885277
+	0	139707	0.843998066815683
+	1 − 5	7018	0.04239714855313236
+	6 − 20	1551	0.009369902736664049
+	21 − 100	981	0.00592641817193258
+	101 − 1000	946	0.005714976137256087
+	> 1000	3910	0.023621095873859722
−	all	11417	0.06897239171147224
−	0	7009	0.04234277774421555
−	1 − 5	1092	0.006596991481906603
−	6 − 20	221	0.0013351054189572886
−	21 − 100	242	0.0014619706397631849
−	101 − 1000	285	0.0017217422823657344
−	> 1000	2568	0.01551380414426388

Table 17 The succesor distances in data set $\mathbf{F}$.

Direction	Distance	Absolute	Fraction
$+$	all	16950668	0.6951258995630639
$+$	0	16046256	0.6580370836488103
$+$	$1-5$	543304	0.022280224103038943
$+$	$6-20$	194421	0.007972964399925151
$+$	$21-100$	44183	0.0018118901048852383
$+$	$101-1000$	21545	$8.835337643381496E-4$
$+$	> 1000	100959	0.004140203542066152
$-$	all	7434365	0.304874100436936
$-$	0	6912515	0.28347367830094794
$-$	$1-5$	285028	0.011688645243990443
$-$	$6-20$	117550	0.004820579902434416
$-$	$21-100$	26756	0.0010972304199875391
$-$	$101-1000$	13254	$5.435301235803125E-4$
$-$	> 1000	79262	0.0032504364459953776

8.5 ORF statistic

ORFs provide an estimate how much biological entities are retained in the correct order. Thus, we calculate how many ORFs are kept together in the final order. These is reasoned by the idea that ORFs should not be fragmented in the process as long the genes have the same biological functions in the species in the alignment. The ORFs are obtained from the NCBI annotations of the reference species. For data set **F**, not the complete ORFs but exons are used.

To measure how fragmented a ORF is, the distances between all adjacent blocks in the ORF are add up to the total size of the gaps between the blocks of an ORF. The gap sizes of the ORFs are then binnede. Furthermore, for each bin, the number of broken ORFs and the number of ORFs with more than one block are counted. Broken means that the relative order of the blocks is not kept in the genome order. Thus, a block exists in the ORF where both adjacent blocks are before it or both are after it in the order.

The results are shown in Table 18, 19, and 20.

Table 18 The distribution of the total gap sizes between blocks of annotated ORF in data set B.

Gap size	Absolute	Fraction	Sum of Fractions	Broken	> 1 Blocks
0	4806	0.9578	0.9578	0	882
$1 - 100$	104	0.0207	0.9785	2	104
> 100	108	0.0215	1.0000	48	108

Table 19 The distribution of the total gap sizes between blocks of annotated ORF in data set Y.

Gap size	Absolute	Fraction	Sum of Fractions	Broken	> 1 Blocks
0	5474	0.9030	0.9030	0	4387
$1 - 100$	272	0.0449	0.9479	2	272
> 100	316	0.0521	1.0000	71	316

Table 20 The distribution of the total gap sizes between blocks of annotated exons in data set F.

Gap size	Absolute	Fraction	Sum of Fractions	Broken	> 1 Blocks
0	158956	0.9515	0.9515	0	107594
$1 - 100$	2387	0.0143	0.9658	161	2387
> 100	5708	0.0342	1.0000	618	5708

8.6 Betweeness statistic

The quality of the result for the Colored Multigraph Betweenness Problem is difficult to measure. To solve this, the results of different method and steps are compared. Three orders are compared or, to be more accurate, the graphs that with the corresponding order are solution to the Colored Multigraph Betweenness Problem. As reference, the original graph is part of the comparison. The vertex set is thus the same for all graphs and only the number of edges and the number of triples that are created out of the graph with the definition in Colored Multigraph Betweenness Problem differs. These are given as a absolute number and as fraction of the original graph. The results are shown in Table 21, 22, and 23.

Table 21 The comparison of different betweenness graphs for data set B.

Graph	Edge number	Fraction	Betweenness triples	Fraction
Orignal graph	35843	1.0000	35835	1.0000
FAS graph	33438	0.9329	31185	0.8702
Before optimization graph	34123	0.9520	32534	0.9079
Betweenness graph	34146	0.9527	32578	0.9091

Table 22 The comparison of different betweenness graphs for data set Y.

Graph	Edge number	Fraction	Betweenness triples	Fraction
Orignal graph	164113	1.0000	158098	1.0000
FAS graph	134567	0.8200	103126	0.6523
Before optimization graph	159911	0.9744	150151	0.9497
Betweenness graph	159996	0.9749	150308	0.9507

Table 23 The comparison of different betweenness graphs for data set F.

Graph	Edge number	Fraction	Betweenness triples	Fraction
Orignal graph	24382316	1.0000	24373551	1.0000
FAS graph	21164222	0.8680	18264760	0.7494
Before optimization graph	24236459	0.9940	24086318	0.9882
Betweenness graph	24237938	0.9941	24089235	0.9883

Comparative analysis of the tricarboxylic acid cycle in yeast

The tricarboxylic acid (TCA) cycle for aerobic respiration is well studied and discussed in yeast (e.g. [10]). In *S. cerevisiae*, 20 genes belong to the TCA cycle [11, 12, 13, 14]. All of them are part of the initial set of alignment blocks in the yeast data set (**Y**). The TCA cycle is at least for *S. cerevisiae* essential. Consequently, we expect that the genes of TCA cycle are conserved in all yeast species in the yeast data set.

All genes are distributed over at least two blocks in *S. cerevisiae*. For all genes, we found evidences that they are contained in the initial alignment also for the other species. Note that we use BLAST to search the protein sequences in the genome assembly since there were no annotations available. Many genes of the TCA cycle are conservered in large parts or completely in all species i.e. the sequences from different species belongs to the same alignment block. Nevertheless, there are serveral cases with low conservation for one species. For example, SDH1 in *S. kudriavzevii* and *S. cerevisiae* do not share any nucleotide between the annotations (based on BLAST) but in the alignment blocks for SDH1 in *S. cerevisiae*, a sequence for *S. kudriavzevii* can be found. However, all those issues are likely artefacts from the assemblies and alignment procedure.

After filtering some blocks are lost. Five genes of all genes loose one block each due to the length and score filter. However, the information loss is negligible since in each case less than 1% of the gene is lost. The overlap filter discards blocks of 11 genes such that large parts or almost complete genes are lost. We investigated those cases more deeply and found that those genes exhibit a high resemblance to either other genes in the TCA cycle or pseudogenes that result from whole genome duplications [15]. Except *S. cerevisiae*, all genome assemblies in the yeast data set consists of contigs and thus have a rather low quality. Thus, very similar nucleotide sequences might be merged into one sequence. As a result very similar (part of) genes may are not assembled as separate genes but occur only one in the assembly. A reliable assignment of those misassemblies to genes is therefore difficult.

In the following cases, the overlap filters discards parts or complete genes. Large parts of IDH1 are lost in most of the species due to high similarity to IDH2. Also a fifth of IDH2 is lost due to this similiarity. The same hold for CIT1 and CIT3 where CIT1 is lost in most of the species and a fifth of CIT3. PYC1 and PYC2 are very similar in sequence. After filtering almost all blocks for those genes are discarded. After filtering, at least 50% of ACO1 is lost due to the similarity to ACO2. Two species loosing ACO1 completely. Large parts of AC02 are retained. SDH1 and its homolog YJL045W is lost almost completely in all species due to there homology. Also SDH3 has a homolog that is a pseudogene: SHH3. This leads to loss of large parts or the complete loss of SDH3 after filtering. Another gene where a part of the gene is filtered is the MDH1 gene. The filtered part overlap with the MDH3 gene. The MDH3 is a very similar gene that has a comparable function as the MDH1 gene in the glyoxylate cycle an other variation of the TCA cycle.

To summarize the results after filtering, the low assembly quality and the alignment method for the whole genome alignment causes the removal of blocks for 11 genes. For further analysis, we will use only the 9 genes which are kept completely after filtering. These 9 genes are almost complete in the same blocks for all species.

For 7 genes, the blocks of the gene are placed as successive blocks with successive genomic coordinates in the optimized order. The block for KGD2 as well as the block for SDH2 are broken apart by the MFAS heuristic. Since the information that they should be successive is lost, they are places in different locations in the optimized order. However, at least within the two parts, blocks and genomic coordinates are successive.

To summarize, the common coordinate system for yeast shows the conservation of 9 of the 20 genes in TCA cycle. Filtering statistics indicate that the remaining 11 genes can not be analyzed with respect to conservation. They have homologs or share sequence similarity with other genes such that it is unclear if the sequence is conserved or not. The raw multiple alignments used as input thus suggest conservation which is questionable. For conserved genes, the method mostly creates the correct order of the alignments block allowing to easily access the corresponding alignment parts for comparative analysis.

Author details
[1]Competence Center for Scalable Data Services and Solutions Dresden/Leipzig, Universität Leipzig, Augustusplatz 12, D-04107 Leipzig, Germany. [2]Bioinformatics Group, Department of Computer Science, Universität Leipzig, Härtelstraße 16–18, D-04107 Leipzig, Germany. [3]Interdisciplinary Center for Bioinformatics, Universität Leipzig, Härtelstraße 16–18, D-04107 Leipzig, Germany. [4]Automatic Language Processing Group, Department of Computer Science, Universität Leipzig, Augustusplatz 12, D-04107 Leipzig, Germany. [5] Max Planck Institute for Mathematics in the Sciences, Inselstraße 22, D-04103 Leipzig, Germany. [6] Fraunhofer Institut for Cell Therapy and Immunology, Perlickstraße 1, D-04103 Leipzig, Germany. [7]Department of Theoretical Chemistry, University of Vienna Währinger Straße 17, A-1090 Vienna, Austria. [8] Center for non-coding RNA in Technology and Health, Grønegårdsvej 3, DK-1870 Frederiksberg C, Denmark. [9] Santa Fe Institute, 1399 Hyde Park Rd., NM87501 Santa Fe, USA.

References

1. Opatrny, J.: Total ordering problem. SIAM J Computing **8**, 111–114 (1979)
2. Paten, B., Earl, D., Nguyen, N., Diekhans, M., Zerbino, D., Haussler, D.: Cactus: Algorithms for genome multiple sequence alignment. Genome research **21**(9), 1512–1528 (2011)
3. Chiaromonte, F., Yap, V., Miller, W.: Scoring pairwise genomic sequence alignments. In: Pacific Symposium on Biocomputing, vol. 7, p. 115 (2001)
4. Karp, R.M.: Reducibility among combinatorial problems. In: Complexity of Computer Computations, pp. 85–103. Springer, ??? (1972)
5. Chen, J., Liu, Y., Lu, S., O'Sullivan, B., Razgon, I.: A fixed-parameter algorithm for the directed feedback vertex set problem. J. ACM **55**, 1–19 (2008)
6. Kann, V.: On the approximability of NP-complete optimization problems. PhD thesis, Royal Institute of Technology, Stockholm (1992)
7. Eades, P., Lin, X., Smyth, W.F.: A fast and effective heuristic for the feedback arc set problem. Inf Processing Let **47**, 319–323 (1993)
8. Saab, Y.: A fast and effective algorithm for the feedback arc set problem. J Heuristics **7**, 235–250 (2001). doi:10.1023/A:1011315014322
9. Kahn, A.B.: Topological sorting of large networks. Communications of the ACM **5**(11), 558–562 (1962)
10. Krebs, H., Gurin, S., Eggleston, L.: The pathway of oxidation of acetate in baker's yeast. Biochemical Journal **51**(5), 614 (1952)
11. Saccharomyces Genome Database Community: SGD Yeast Pathway: Saccharomyces cerevisiae TCA cycle, aerobic respiration. http://pathway.yeastgenome.org/YEAST/NEW-IMAGE?object=TCA-EUK-PWY. Accessed: 2017-05-18
12. Haselbeck, R.J., McAlister-Henn, L.: Function and expression of yeast mitochondrial nad-and nadp-specific isocitrate dehydrogenases. Journal of Biological Chemistry **268**(16), 12116–12122 (1993)
13. Oyedotun, K.S., Lemire, B.D.: The carboxyl terminus of the saccharomyces cerevisiaesuccinate dehydrogenase membrane subunit, sdh4p, is necessary for ubiquinone reduction and enzyme stability. Journal of Biological Chemistry **272**(50), 31382–31388 (1997)
14. Yasutake, Y., Watanabe, S., Yao, M., Takada, Y., Fukunaga, N., Tanaka, I.: Crystal structure of the monomeric isocitrate dehydrogenase in the presence of nadp+ insight into the cofactor recognition, catalysis, and evolution. Journal of Biological Chemistry **278**(38), 36897–36904 (2003)
15. Byrne, K.P., Wolfe, K.H.: The yeast gene order browser: combining curated homology and syntenic context reveals gene fate in polyploid species. Genome research **15**(10), 1456–1461 (2005)
